# USP13 regulates the RAP80-BRCA1 complex dependent DNA damage response

**DOI:** 10.1038/ncomms15752

**Published:** 2017-06-01

**Authors:** Yunhui Li, Kuntian Luo, Yujiao Yin, Chenming Wu, Min Deng, Lei Li, Yuping Chen, Somaira Nowsheen, Zhenkun Lou, Jian Yuan

**Affiliations:** 1 Research Center for Translational Medicine, East Hospital, Tongji University School of Medicine, Shanghai 200120, China; 2 Key Laboratory of Arrhythmias of the Ministry of Education of China, East Hospital, Tongji University School of Medicine, Shanghai 200120, China; 3 Department of Oncology, Mayo Clinic, Rochester, Minnesota 55905, USA; 4 Medical Scientist Training Program, Mayo Clinic School of Medicine, Mayo Clinic Graduate School of Biomedical Sciences, Rochester, Minnesota 55905, USA

## Abstract

BRCA1 regulates multiple cellular pathways that maintain genomic stability including cell cycle checkpoints, DNA repair, protein ubiquitination, chromatin remodelling, transcriptional regulation and apoptosis. Receptor-associated protein 80 (RAP80) helps recruit BRCA1 to double-strand breaks (DSBs) through the scaffold protein CCDC98 (Abraxas) and facilitates DNA damage response (DDR). However, the regulation of RAP80-BRCA1 complex is still unclear. Here we report that a deubiquitinase, USP13, regulates DDR by targeting RAP80. Mechanistically, USP13 is phosphorylated by ATM following DNA damage which, in turn, facilitates its DSB localization. USP13, in turn, deubiquitinates RAP80 and promotes RAP80 recruitment and proper DDR. Depleting or inhibiting USP13 sensitizes ovarian cancer cells to cisplatin and PARP inhibitor (olaparib) while overexpression of USP13 renders ovarian cancer cells resistant to chemotherapy. Overall, we identify USP13 as a regulator of DNA repair and reveal a model in which a phosphorylation-deubiquitination axis dynamically regulates RAP80-BRCA1 complex foci formation and function.

## Abstract (web summary)

RAP80 helps to recruit BRCA1 to double-strand breaks, facilitating DNA damage responses. Here the authors report that phosphorylated USP13 deubiquitinates RAP80 after DNA damage, prompting recruitment to the break site.

Maintenance of genomic stability is critical for the wellbeing of organisms^1^,^2^. The genome of a cell is under constant attack from exogenous and endogenous DNA damaging agents such as radiation, carcinogens and reactive radicals^3^. To maintain genomic stability, cells have developed an elaborate DNA damage response (DDR) system which is responsible for sensing DNA damage, halting the ongoing cell cycle and repairing the damaged DNA^4^,^5^,^6^,^7^,^8^. Failure in the DDR pathway leads to genomic instability which is one of the driving forces of tumorigenesis^4^,^9^.

BRCA1 functions in a number of cellular pathways that maintain genomic stability including DNA damage-induced cell cycle checkpoint activation, DNA repair, protein ubiquitination, chromatin remodelling, transcriptional regulation and apoptosis^10^. One of the major functions of BRCA1 in DDR is to promote homologous recombination (HR). BRCA1 is recruited to double-strand breaks (DSBs) through a cascade of signal transduction events. Following DNA damage, chromatin-associated histone H2AX is phosphorylated by ATM and ATR and subsequently recruits a phospho-module-binding mediator MDC1 (refs 11, 12, 13). Downstream of MDC1, the E3 ligases RNF8 and RNF168 are recruited^14^,^15^,^16^,^17^,^18^,^19^ resulting in ubiquitination of histones and other factors at the sites of DNA damage. The ubiquitin (Ub) chains act as docking sites for RAP80 through its ubiquitin-interacting motifs (UIMs). RAP80 further recruits BRCA1 to the DSBs through the scaffold protein CCDC98 (refs 20, 21, 22). Previous studies suggested RAP80 is important for BRCA1 recruitment to the DSBs and RAP80 depletion results in loss of BRCA1 focus formation, induces genomic instability and impairs HR^20^,^21^,^22^. Others also propose that RAP80 fine tunes BRCA1 activity and prevents excessive HR by blocking DSB end resection^23^,^24^. BRCA1 forms complexes with different DNA damage factors, such as BACH1, CtIP, BRCA2 and Rad51 (ref. 25). All these complexes are recruited to DNA damage sites and are important for proper DDR^5^. For example, the BRCA1-CtIP complex, in conjunction with the MRN complex, mediates extensive DSB end resection that generates single-strand DNA (ssDNA) overhangs to support subsequent HR-mediated repair of DSB^26^; BRCA1-BRCA2-Rad51 complex plays a major role in homology search during HR^27^.

DSB-induced BRCA1 focus formation is essential for proper BRCA1 function in DDR. To further study the regulation of BRCA1 focus formation, we performed a targeted shRNA library screen of the USP subfamily of deubiquitinases for their role in regulating BRCA1 focus formation. We found that USP13 plays an important role in DNA repair and one of the components of the BRCA1 complex, RAP80, is a target of USP13. In response to DNA damage, USP13 is phosphorylated by ATM, which in turn facilitates USP13 recruitment to DSBs, RAP80 deubiquitination, and triggers DDR signalling. Importantly, depleting or inhibiting USP13 sensitizes ovarian cancer cells to cisplatin and Poly (ADP-ribose) polymerase (PARP) inhibitor (olaparib) suggesting USP13 is a potential novel therapeutic target for ovarian cancer.

## Results

### USP13 regulates RAP80-BRCA1 complex foci formation

**Figure 1:**
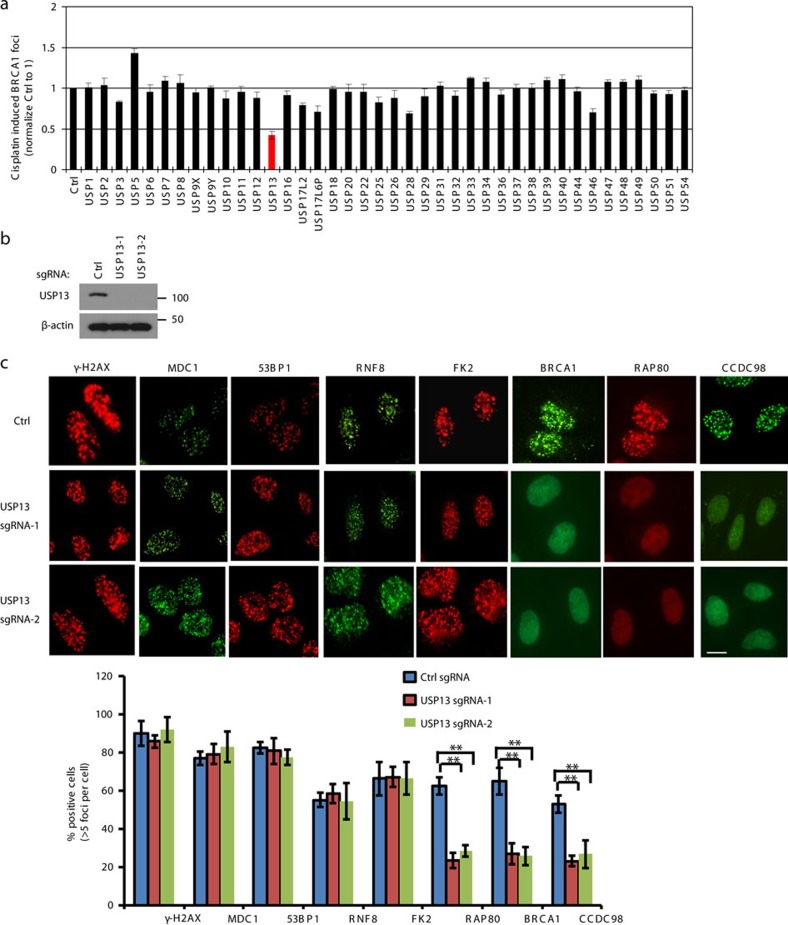
USP13 regulates RAP80-BRCA1 foci formation. (**a**) A panel of deubiquitinases was knocked down in EFO-27 cells and cisplatin-induced BRCA1 foci were assayed. (**b**) USP13 was knocked out in EFO-27 cells by CRISPR and western blot was performed with the indicated antibodies. (**c**) Control or USP13 knockout EFO-27 cells were treated with cisplatin and foci formation of the indicated factors were detected by immunofluorescence with indicated antibodies. Representative images are shown in the upper panels. Scale bar, 10 μm. Quantification of the percentage of cells displaying foci formation is shown in the lower panels. (**a**,**c**) Error bars represent±s.e.m. from three independent experiments. **P<0.01. More than 200 cells were counted per experiment. Statistical analyses were performed with the ANOVA. ANOVA, analysis of variance.

DSB-induced BRCA1 focus formation is one of the key signatures for proper BRCA1 function in the DDR^27^. We performed a targeted shRNA library screen of USP family of deubiquitinases for their role in regulating BRCA1 focus formation. We found that knockdown of USP13 dramatically decreased cisplatin-induced BRCA1 foci formation (Fig. 1a). USP13 is a deubiquitination enzyme that has been implicated in melanocyte development, hypoxia signalling, tumorigenesis and the endoplasmic reticulum stress pathway^28^,^29^,^30^,^31^,^32^. A previous publication reported that USP13 regulates PTEN^31^. To exclude the probable role of PTEN in the DDR, we chose the PTEN-null BRCA1 wild-type (WT) ovarian cancer cell line EFO-27 as our cell model. To confirm the role of USP13 in regulating BRCA1 focus formation, we generated USP13-null EFO-27 cells using the CRISPR-Cas9 system and confirmed that knockout of USP13 reduces BRCA1 but not 53BP1 focus formation (Fig. 1b,c). Interestingly, RAP80 and CCDC98, two components of the BRCA1-A complex regulating BRCA1 focus formation, also could not form foci. However, focus formation of γ-H2AX, MDC1, RNF8 and FK2 (Ub), the upstream regulators of RAP80, did not change (Fig. 1c). We obtained similar results in U2OS and HeLa cells (Supplementary Fig. 1a-b). In addition, knockout of USP13 reduced foci formation and chromatin association of RAP80-BRCA1 complex in response to IR and PARP inhibitor treatment (Supplementary Fig. 2a-c). Furthermore, USP13 deficiency decreased BRCA1 recruitment to DSBs generated by I-SceI (Supplementary Fig. 2d). To evaluate the recruitment of USP13 and RAP80 at DSBs, we analysed the kinetics of GFP-USP13 and GFP-RAP80 accumulation on stripes after laser microirradiation. Notably, we observed that the accumulation of GFP-USP13 at DNA-damage sites peaked around 60 s, while GFP-RAP80 peaked ∼90 s (Supplementary Fig. 3a-b), suggesting USP13 is recruited earlier than RAP80.

Taken together, these results suggest that USP13 regulates the focus formation of the RAP80-BRCA1 complex. Since RAP80 recruits BRCA1 to DSBs through CCDC98, we hypothesized that RAP80 might be the target of USP13.

### USP13 interacts with and deubiquitinates RAP80

**Figure 2:**
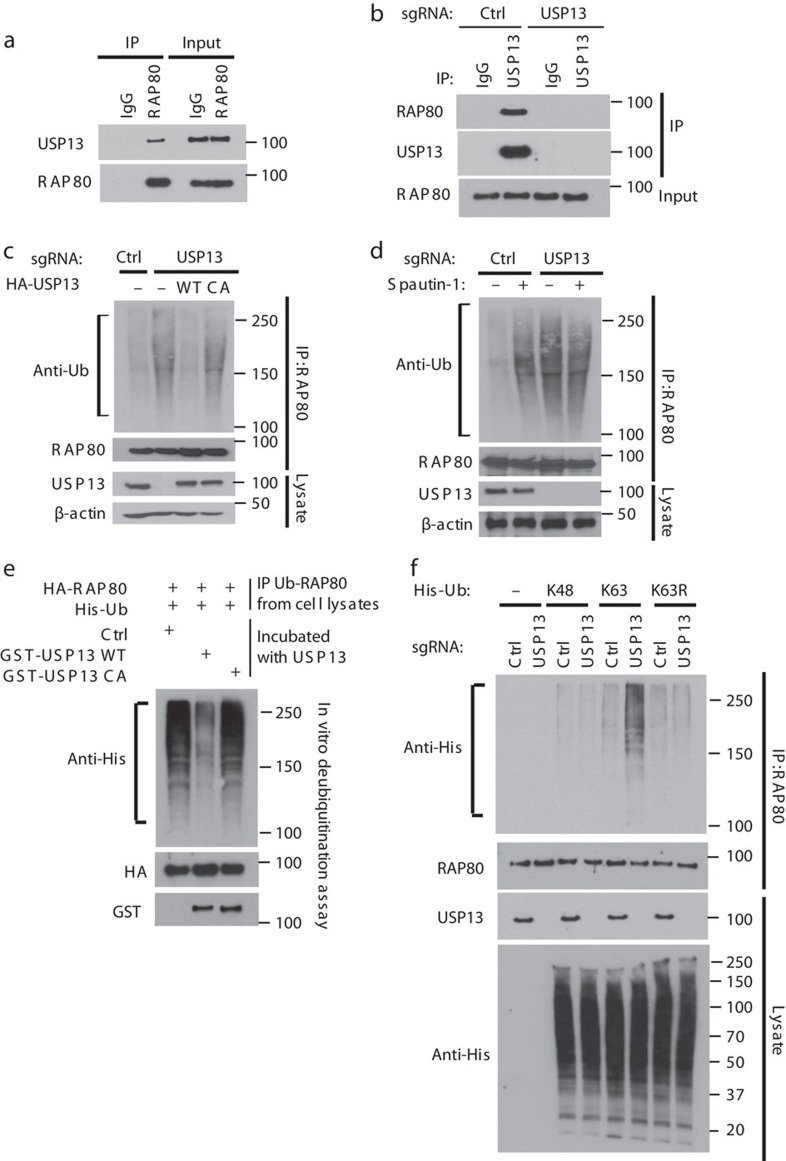
USP13 interacts with and deubiquitinates RAP80. (**a**) EFO-27 cell lysates were subjected to immunoprecipitation with control IgG or RAP80 antibodies. The immunoprecipitates were then blotted with the indicated antibodies. (**b**) Cell lysates from control (Ctrl) or USP13 knockout cells were subjected to immunoprecipitation with control IgG or USP13 antibodies. The western blots were then blotted with the indicated antibodies. (**c**) Control, USP13 knockout, and USP13 knockout cells stably expressing the indicated constructs were lysed under denaturing conditions and RAP80 was immunoprecipitated. Blots were probed with the indicated antibodies. (**d**) Control and USP13 knockout cells treated with or without Spautin-1 were lysed under denaturing conditions and RAP80 was immunoprecipitated. Blots were probed with the indicated antibodies. (**e**) Ubiquitinated HA-RAP80 was incubated with purified USP13 or USP13 CA *in vitro* and then blotted with the indicated antibodies. (**f**) Control or USP13 knockout cells were transfected with indicated His-Ub lysine-specific mutant constructs, cells were lysed under denaturing conditions and RAP80 was immunoprecipitated. Blots were probed with the indicated antibodies.

To test our hypothesis, we first examined whether USP13 interacts with RAP80. We found that endogenous RAP80 co-immunoprecipitated with USP13 (Fig. 2a). Reciprocal immunoprecipitation with USP13 antibody also brought down RAP80 in control but not USP13-deficient cells (Fig. 2b). Since USP13 is an ubiquitin-specific protease that functions to remove ubiquitin chains from its substrate proteins, we analysed the effect of USP13 expression on RAP80 ubiquitination. As shown in Fig. 2c, USP13 deficiency dramatically elevated the ubiquitination of RAP80 *in vivo.* Reconstituting with USP13-WT, but not the USP13- catalytically inactive (CA) mutant in USP13 knockout cells reversed the increase in RAP80 ubiquitination induced by USP13 deficiency and rescued RAP80 foci formation following DNA damage (Fig. 2c and Supplementary Fig. 4a-c). Interestingly, RAP80 protein levels and the levels of other factors in the BRCA1-A complex did not change when we modulated USP13 expression (Fig. 2c,d and Supplementary Fig. 4d). In addition, USP13 specific inhibitor, Spautin-1 (ref. 28), significantly enhanced RAP80 ubiquitination in control but not USP13-deficient cells (Fig. 2d). Collectively, these results suggest that USP13 regulates RAP80 ubiquitination and foci formation through its deubiquitinase activity. Next, we performed an *in vitro* deubiquitination assay to further confirm that USP13 directly deubiquitinates RAP80. We found that incubation of ubiquitinated RAP80 with purified WT USP13, but not with the USP13 CA mutant, significantly decreases RAP80 ubiquitination *in vitro* (Fig. 2e). As shown in Fig. 2f, RAP80 can be conjugated with both K48-linked and K63-linked ubiquitin chains. However, knocking out USP13 dramatically increased K63-linked ubiquitin chain but not the K48-linked of RAP80, suggesting that USP13 deubiquitinates K63-linked ubiquitin chain of RAP80. Taken together, these results suggest that USP13 deubiquitinates RAP80 both *in vitro* and *in vivo*.

### USP13 regulates DNA damage response through RAP80

**Figure 3:**
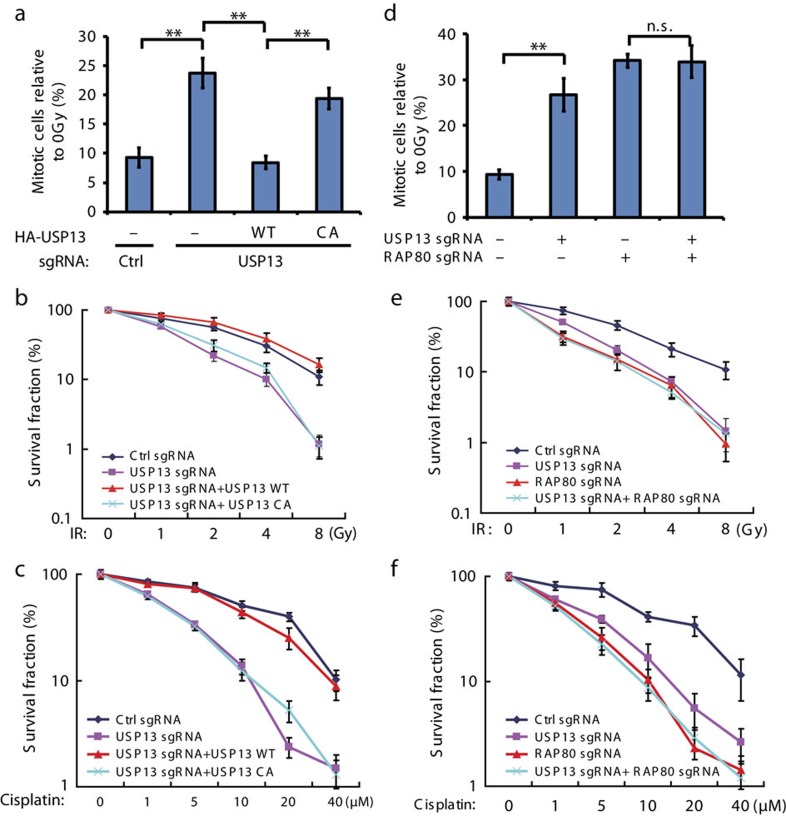
USP13 regulates DDR and radiosensitivity through RAP80. (**a**) Control, USP13 knockout, and USP13 knockout cells stably expressing the indicated constructs were left untreated or treated with IR (2 Gy). After a further 1 h, cells were collected, fixed and stained with anti-phospho-H3 antibodies to determine the mitotic population (mitotic index). (**b**-**c**) The sensitivity of the same cells as in **a** to IR (**b**) and cisplatin (**c**) were assessed using colony formation assay. (**d**) Control and USP13 knockout, RAP80 knockout or USP13 and RAP80 double-knockout cells were subjected to mitotic population determination as in **a**. (**e**–**f**) The sensitivity of cells the same as in **d** to IR (**e**) and cisplatin (**f**) were assessed using colony formation assay. (**a**-**f**) Error bars represent±s.e.m. from three independent experiments. **P<0.01. Statistical analyses were performed with the ANOVA. ANOVA, analysis of variance.

The RAP80-BRCA1 pathway plays an important role in DNA repair and DNA damage signal transduction^20^,^21^,^22^. Therefore, we tested whether USP13 is involved in DNA repair, cell cycle checkpoint, response to radiation and chemo sensitivity. As shown in Fig. 3a-c and Supplementary Fig. 5a-c, knocking out USP13 compromised the DNA damage-induced G2/M checkpoint and sensitized cells to irradiation and cisplatin treatment. Reconstituting USP13 knockout cells with USP13-WT, but not the USP13-CA mutant, rescued these phenotypes. To further confirm that USP13 regulated DDR through RAP80, we generated RAP80 knockout cells by CRISPR-cas9. As shown in Fig. 3d-f and Supplementary Fig. 5d, RAP80-deficiency compromised the DNA damage-induced G2/M checkpoint and sensitized cells to irradiation and cisplatin treatment. Furthermore, loss of USP13 had no further effect on DNA damage-induced G2/M checkpoint and radiation and chemo sensitivity in RAP80 deficient cells, suggesting that USP13 regulates DDR by targeting RAP80. In addition, we found that USP13 deficiency results in decreased HR (Supplementary Fig. 6a). USP13 inhibitor, Spautin-1, treatment also impaired HR in control but not USP13-deficient cells (Supplementary Fig. 6b). Taken together, these results suggest that USP13 regulates DDR through RAP80.

### Deubiquitination of RAP80 by USP13 is important for DDR

**Figure 4:**
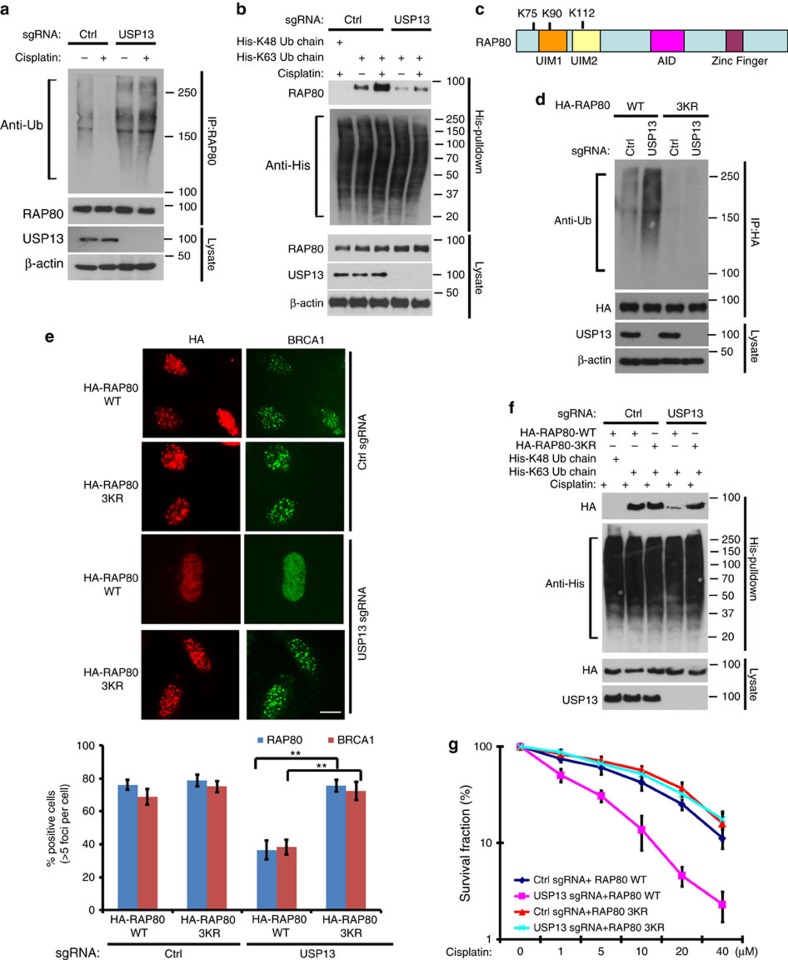
Deubiquitination of RAP80 by USP13 is important for RAP80-BRCA1 foci formation and DDR. (**a**) Control and USP13 knockout cells treated with or without cisplatin were lysed under denaturing conditions and RAP80 was immunoprecipitated. Blots were probed with the indicated antibodies. (**b**) Control and USP13 knockout cells treated with or without cisplatin were lysed and cell lysates were subjected to pull-down assay by incubating with indicated His-Ub chains conjugated with Ni-NTA beads. After washing, proteins bound to beads were subjected to western blot with indicated antibodies. (**c**–**d**) Control and USP13 knockout cells transfected with HA-RAP80 WT or 3KR constructs were lysed under denaturing conditions and HA-RAP80 was immunoprecipitated. Blots were probed with the indicated antibodies. (**e**) Control or USP13 knockout cells transfected with WT or 3KR HA-RAP80 were treated with cisplatin. RAP80 and BRCA1 foci formation were detected by immunofluorescence. Scale bar, 10 μm. Error bars represent±s.e.m. from three independent experiments. **P<0.01. More than 200 cells were counted per experiment. Statistical analyses were performed with the ANOVA. (**f**) Control or USP13 knockout cells transfected with WT or 3KR HA-RAP80 were lysed and cell lysates were subjected to pull-down assay as in **b**. (**g**) Control or USP13 knockout cells transfected with control vector, WT or 3KR HA-RAP80 were subjected to colony formation assay to assess the sensitivity of cells to cisplatin. Error bars represent±s.e.m. from three independent experiments. ANOVA, analysis of variance.

So far, we show that USP13 is important for RAP80 recruitment to DSBs and DDR (Figs 1 and 3). Consistent with this, we found that RAP80 ubiquitination decreases following DNA damage (Fig. 4a and Supplementary Fig. 7a), suggesting that USP13 promotes RAP80 deubiquitination following DNA damage. Together with results showing USP13 is important for DDR and RAP80 localization at the sites of DNA damage, we hypothesized that RAP80 ubiquitination is inhibitory of its function, and deubiquitination of RAP80 by USP13 following DNA damage promotes RAP80 function in the DDR pathway. It is well established that the UIM domains of RAP80 bind with polyubiquitin K63 linkage following DNA damage, which is critical for the recruitment of RAP80 to the DNA damage sites. We hypothesized that USP13 may regulate RAP80 binding with polyubiquitin K63-linkage following DNA damage through deubiquitinating RAP80. As shown in Fig. 4b, RAP80 specifically interacted with K63-linked polyubiquitin chain, which was dramatically increased following cisplatin treatment in USP13 proficient cells. Interestingly, in USP13-deficient cells, the basal RAP80-K63-linked polyubiquitin chain interaction was significantly compromised (Fig. 4b). These results suggest that deubiquitination of RAP80 by USP13 following DNA damage facilitates the binding between RAP80 and K63- linked polyubiquitin chain.

Next, we mapped potential ubiquitination sites of RAP80 that are targeted by USP13. Previous studies suggested that RAP80 binds to polyubiquitin chains through its UIM domains. After analysing the publicly available proteomics database (https://www.nextprot.org/entry/NX_Q96RL1/proteomics), we found 15 sites on RAP80 that may be ubiquitinated: K20, K31, and K75 at the N-terminal domain, K90 and K112 at the UIM1 and UIM 2 domain and K146, K232, K245, K374, K382, K544, K567, K587, K607 and K635. We found that mutating the 3 sites (K75, K90 and K112, 3KR) near or at the UIM domains, but not the other sites, abolished USP13 deficiency-induced RAP80 ubiquitination (Fig. 4c,d and Supplementary Fig. 7b-c). In addition, we found all the single mutations of these three sites cannot abolish the USP13 deficiency-induced RAP80 ubiquitination (Supplementary Fig. 7d). These results suggest that K75, K90 and K112 of RAP80 are key deubiquitination sites targeted by USP13. Next, we further explored how RAP80 deubiquitination regulates its localization and function. First, we found both recombinant RAP80 WT and 3KR were able to bind to polyubiquitin chains *in vitro,* suggesting that 3KR mutant did not affect RAP80 structure (Supplementary Fig. 7e). Next, we examined the focus formation of RAP80 using WT RAP80 and 3KR mutant. As shown in Fig. 4e, both WT RAP80 and the 3KR mutant form foci normally in USP13 proficient cells. In USP13-deficient cells, however, WT RAP80 focus formation was compromised, which in turn inhibited BRCA1 recruitment to DSBs. Interestingly, 3KR mutation rescued RAP80 and BRCA1 foci formation in USP13-deficient cells. In addition, USP13 deficiency resulted in decreased binding between RAP80 and K63-linked ubiquitin chain; however, it had no effect on the ability of the 3KR mutant to bind K63-linked ubiquitin chain (Fig. 4f). Finally, we found that the 3KR mutant but not WT RAP80 was capable of rescuing cell viability in response to cisplatin in USP13-deficient cells (Fig. 4g). Taken together, these results demonstrated that ubiquitination of RAP80 around its UIM domain interferes with its interaction with polyubiquitin. Deubiquitination of RAP80 by USP13 plays an important role in the ability of RAP80 to bind polyubiquitin, which is important for RAP80 recruitment and DDR.

### USP13 is phosphorylated by ATM following DNA damage

**Figure 5:**
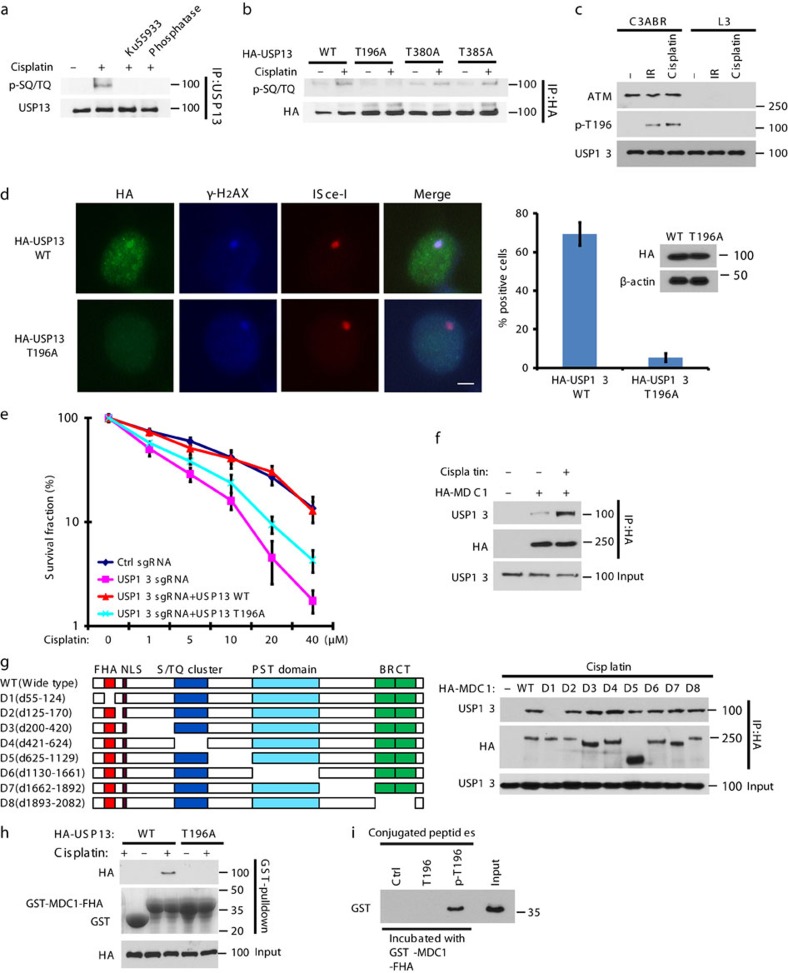
Regulation of the DDR signalling by USP13. (**a**) HEK293T cells were pretreated with DMSO or 25 μM Ku55933 for 2 h following which they were left untreated or treated with cisplatin. After an additional 1 h, USP13 was immunoprecipitated, left untreated or treated with phosphatase, and immunoblotted with phospho-SQ/TQ (pSQ/TQ) antibody. (**b**) HEK293T cells transfected with indicated constructs were left untreated or treated with cisplatin. HA-USP13 was immunoprecipitated and blotted with phospho-SQ/TQ (pSQ/TQ) antibody. (**c**) ATM-proficient cells (C3ABR) and ATM-deficient cells (L3) were treated as indicated. After 1 h, USP13 was immunoprecipitated and blots probed with the antibody specifically recognizing phosphorylation of USP13 at T196. (**d**) Co-localization of WT or T196A USP13 with γ-H2AX at DSB site created by I-SceI. Positive staining cells are quantified in the right panel. Scale bar, 5 μm. Error bars represent±s.e.m. from three independent experiments. >100 cells were counted per experiment. (**e**) Control, USP13 knockout, and USP13 knockout cells stably expressing the indicated constructs were subjected to colony formation assay to assess the sensitivity of cells to cisplatin. Error bars represent±s.e.m. from three independent experiments. (**f**) HEK293T cells transfected with HA-MDC1 were left untreated or treated with cisplatin. Cell lysates were subjected to immunoprecipitation with HA antibody. The immunoprecipitates were then blotted with the indicated antibodies. (**g**) HEK293T cells transfected with deletion mutants of HA-MDC1 were subjected to co-immunoprecipitation as in **f**. (**h**) HEK293T cells transfected with WT or T196A USP13 were treated with cisplatin, and cell lysates were incubated with Sepharose coupled with GST or GST-MDC1-FHA domain. After washing, proteins bound on Sepharose were blotted with the indicated antibodies. (**i**) Nonphosphorylated or phosphorylated Thr 196 peptide (T196 or p-T196, respectively) was conjugated to Sepharose beads and incubated with purified GST-MDC1-FHA domain in NETN buffer. After washing, proteins bound to beads were blotted with the indicated antibodies.

Our results suggest that USP13 deubiquitinates RAP80 following DNA damage, which in turn facilitates RAP80 recruitment to DSBs. However, how USP13 is regulated in response to DNA damage is unclear. We found that following cisplatin treatment, USP13 is phosphorylated at SQ/TQ motifs (Fig. 5a and Supplementary Fig. 8a-b), which are consensus ATM or ATR phosphorylation sites^33^. Pretreating cells with Ku55933, a specific ATM inhibitor, or treating cell lysates with lambda phosphatase, abolished this phosphorylation. This result suggests that USP13 may be phosphorylated by ATM following DNA damage. We next analysed the USP13 protein sequence and found three threonine sites fitting the ATM consensus phosphorylation motif (SQ/TQ motif): T196, T380 and T385. We further examined the phosphorylation of these candidate sites and found that the T196A mutation abolished the phosphorylation detected by p-SQ/TQ antibody following cisplatin treatment (Fig. 5b). To further confirm these results, we generated site-specific antibody against p-Thr196 and examined the USP13 phosphorylation following DNA damage. As shown in Supplementary Fig. 8c, T196 was phosphorylated after DNA damage, and the T196A mutation inhibited USP13 phosphorylation after DNA damage. In addition, USP13 was phosphorylated on Thr196 following DNA damage in ATM-proficient cells but not ATM-deficient cells (Fig. 5c), suggesting ATM phosphorylates USP13 on T196 following DNA damage.

To further characterize the biological significance of USP13 phosphorylation, we stably expressed USP13 WT and T196A mutant in USP13-deficient cells. First, we examined whether USP13 is recruited to the DSB site. We used I-SceI to generate a DSB in cells^34^ and examined USP13 localization. As shown in Fig. 5d, USP13 localized to DSBs, and co-localized with γ-H2AX. Interestingly, T196A mutation abolished its DSB localization (Fig. 5d). Furthermore, reconstitution of USP13 WT but not the T196A fully reversed the increase in RAP80 ubiquitination induced by USP13 deficiency (Supplementary Fig. 8d), suggesting USP13 phosphorylation by ATM is important for RAP80 deubiquitination. In addition, reconstitution of WT USP13, but not the T196A mutant, fully rescued the foci formation of the BRCA1-A complex, DNA repair and cell cycle checkpoint in USP13-deficient cells and reversed hypersensitivity to cisplatin or olaparib induced by USP13 deficiency (Fig. 5e and Supplementary Fig. 8e-h). Together, these results suggest that USP13 phosphorylation by ATM is important for its recruitment to DSBs, DDR and chemo sensitivity.

Next, we investigated how USP13 phosphorylation regulates its localization. Interestingly, we found that depletion of the DNA damage mediator protein MDC1 abolished USP13 recruitment to DSBs (Supplementary Fig. 8i). In addition, USP13 interacted with MDC1 and the interaction between USP13 and MDC1 was dramatically increased following cisplatin treatment (Fig. 5f). Moreover, we examined the interaction between USP13 and different deletion mutants of MDC1 and found that deletion of FHA domain of MDC1 abolishes the USP13-MDC1 interaction (Fig. 5g). To further demonstrate that the FHA domain of MDC1 is critical for USP13-MDC1 interaction, we purified GST-MDC1-FHA fragment from bacteria and performed pull-down assay. As shown in Fig. 5h, MDC1-FHA pulled down WT USP13 but not the T196A mutant following DNA damage. To further demonstrate the direct interaction between the MDC1 FHA domain and phosphorylated Thr 196 of USP13, T196 or p-T196 peptide was incubated with purified GST-MDC1 FHA domain *in vitro*. As shown in Fig. 5i, only the p-T196 peptide bound to the MDC1 FHA domain, suggesting direct binding between p-T196 residue and the MDC1 FHA domain. Taken together, these results demonstrate that USP13 phosphorylation by ATM is important for USP13 recruitment to DSB sites following DNA damage and proper DDR.

### The role of USP13 in chemoresistance

**Figure 6:**
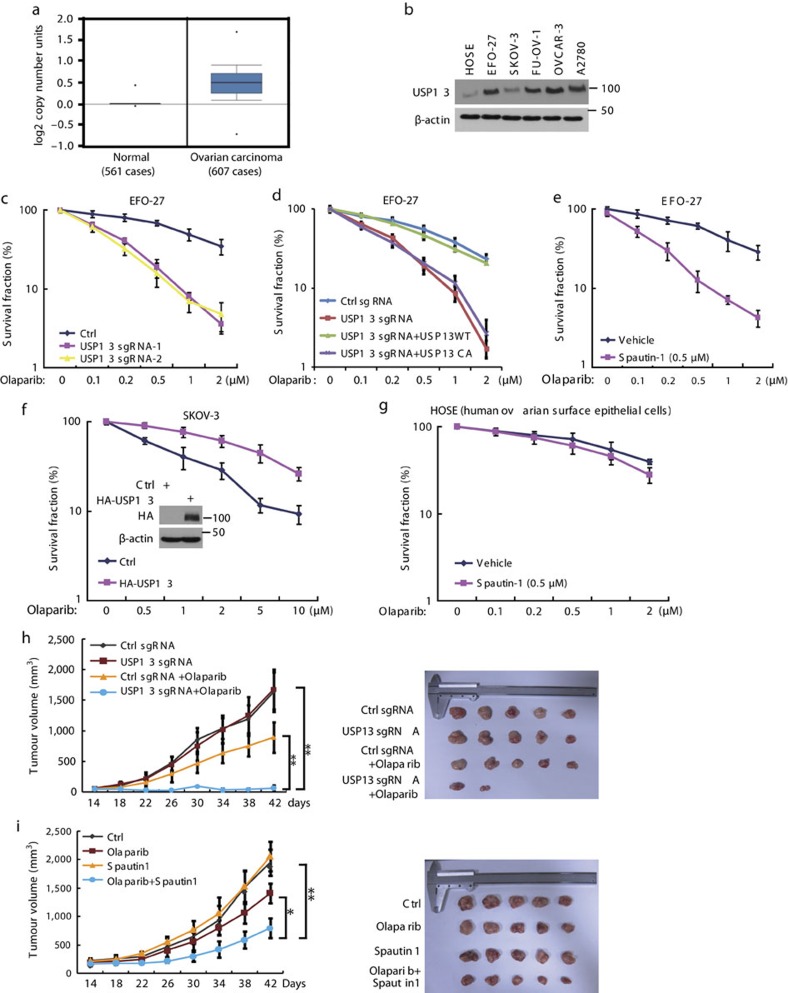
The role of USP13 in response to PARP inhibition. (**a**) USP13 expression in normal tissue and ovarian carcinoma (Oncomine data). (**b**) Expression of USP13 in human ovarian epithelial cell line and ovarian carcinoma cell lines. (**c**) Survival assays for control and USP13 knockout EFO-27 cells exposed to Olaparib. (**d**) Survival assays for control, USP13 knockout, and USP13 knockout cells stably expressing the indicated constructs exposed to olaparib. (**e**) Survival assays for EFO-27 cells exposed to Olaparib or Olaparib together with Spautin-1. (**f**) Survival assays for control and USP13 overexpression SKOV-3 cells exposed to Olaparib. (**g**) Survival assays for HOSE (human ovarian surface epithelial cells) cells exposed to Olaparib or Olaparib together with Spautin-1. (**h**) Tumour growth assay for control and USP13 knockout cells treated with or without Olaparib. (**i**) Tumour growth assay for cells treated with vehicle, Olaparib, Spautin-1 or Olaparib together with Spautin-1. (**c**–**g**) Error bars represent±s.e.m. from three independent experiments. (**h**–**i**) Error bars represent mean s.d. (*n*=5). **P<0.01, *P<0.05. Statistical analyses were performed with the ANOVA. ANOVA, analysis of variance.

Chemoresistance is one of main obstacles for cancer therapeutics. One of the major mechanisms that contributes to cancer chemoresistance is enhanced DDR^35^. For example, platinum-based chemotherapy induces cancer cell apoptosis by generating interstrand crosslinks, which can be adequately repaired by HR-based DNA repair^36^. To escape killing, the tumour cells may evolve to have higher repair efficiency. For example, overexpression of Rad51, which in turn elevates DNA repair capability, renders cells resistant to platinum-based treatment^37^. Since USP13 deubiquitinates RAP80 and regulates DDR, we next examined the role of USP13 in cancer. By surveying a public gene expression database (http://www.oncomine.org), we found that USP13 is upregulated in human ovarian carcinoma (Fig. 6a). We also examined the expression of BRCA-A complex. While BRCA1 and CCDC98 are downregulated in ovarian cancer, RAP80, MERIT40 and BRCC45 are slightly amplified in ovarian cancer, but not comparable to USP13 amplification (Fig. 6a and Supplementary Fig. 9a). We also analysed the BRCA1-A complex in TCGA database. As shown in Supplementary Fig. 9b, 28% ovarian cancers show amplification of USP13, which is higher than other BRCA1-A complex members. Most importantly, although BRCA1 deletion and mutation are observed in a subset of ovarian cancers, they are mostly absent in samples with USP13 amplification. In addition, compared to human ovarian surface epithelial (HOSE) cells, USP13 is overexpressed in ovarian cancer cell lines (Fig. 6b). RAP80 ubiquitination is also decreased in these cell lines (Supplementary Fig. 10a). However, PTEN, whose stability is regulated by USP13 (ref. 31) was also downregulated in these USP13-high ovarian cancer cell lines (Supplementary Fig. 10b), suggesting that in ovarian cancer, PTEN might be downregulated in USP13-independent manner. Next, we examined the role of USP13 in response to chemotherapy. As shown in Fig. 6c,d, knocking out USP13 in EFO-27 cells rendered cells sensitive to the PARP inhibitor, Olaparib. Furthermore, treatment with USP13 inhibitor Spautin-1 also rendered EFO-27 cells sensitive to Olaparib (Fig. 6e). A previous study reported that Spautin-1 also inhibits USP10 activity^28^. However, knocking down USP10 did not sensitize cells to PARPi (PARP inhibitor) or Spautin-1 and PARPi combination treatment (Supplementary Fig. 10c). In addition, Spautin-1 treatment rendered cells sensitive to PARPi in USP10 depleted cells but not in USP13-depleted cells (Supplementary Fig. 10c). These results suggest that USP13 but not USP10 may be the target of Spautin-1 in its regulation of PARPi sensitivity. Conversely, we found that overexpression of USP13 in USP13-low SKOV-3 cells rendered cells resistant to Olaparib (Fig. 6f). Interestingly, Spautin-1 treatment didn't further augment response of HOSE cells with lower expression level of USP13 to Olaparib (Fig. 6g). These results suggest that USP13 may be a good therapeutic target for ovarian cancer with WT BRCA1. Next, we further confirmed the role of USP13 in response to chemotherapy *in vivo*. Knocking out USP13 did not affect cancer cell growth *in vitro* and *in vivo* (Fig. 6h and Supplementary Fig. 10d). In addition, we did not find any changes in cell cycle profile when we deleted *USP13* gene (Supplementary Fig. 10e). However, USP13 deficiency conferred tumours hypersensitive to Olaparib treatment in a xenograft model (Fig. 6h). In addition, compared to single agent treatment with Olaparib, the combination of Spautin-1 and Olaparib showed superior response in xenograft models of ovarian cancer (Fig. 6i). Taken together, our results demonstrate that USP13 may be a causal factor and a therapeutic target of cancer cell response to chemotherapy.

## Discussion

RAP80 forms a complex with BRCA1, CCDC98 (Abraxas), BRCC36 and MERIT40, which was named BRCA1-A complex and plays an important role in DDR^3^,^38^. RAP80 contains tandem UIM domains at its N-terminus and tandem zinc finger domains at its C-terminus^39^. The UIM domains of RAP80 bind with ubiquitin K63 linkages *in vitro* and *in vivo* following DNA damage, which is important for itself and other BRCA1-complex proteins' recruitment to DSBs^20^,^21^,^22^,^40^. However, whether and how RAP80 itself is regulated following DDR is still unclear. We found that following DNA damage, a deubiquitinase, USP13, deubiquitinates RAP80 and promotes binding between RAP80 and K63-linked polyubiquitin chains, which is important for the recruitment of the RAP80-BRCA1 complex to DSBs to facilitate DDR.

RAP80 regulates multiple aspects of DDR. For example, depletion of RAP80 in cells impairs irradiation (IR)-induced CHK1 activation^22^, which causes a defect in the G2/M checkpoint control^20^,^21^,^22^. RAP80 depletion also renders cells sensitive to IR treatment^20^,^21^,^22^,^40^. Moreover, RAP80 plays a role in promoting optimal HR^20^,^40^. However, it was proposed that RAP80 fine tunes BRCA1 activity and prevents excessive HR through blocking DSB end resection^23^,^24^. Our results and other studies suggest that deletion of RAP80 impaired HR^20^,^40^,^41^, suggesting that RAP80's function in HR is context-dependent. Furthermore, we report that USP13 deficiency compromises DNA damage-induced G2/M checkpoint and sensitizes cells to IR and cisplatin treatment in a RAP80-dependent manner.

Overall, this report reveals a new regulatory mechanism for activating RAP80-BRCA1 complex. Besides the UIM domains, RAP80 also contains a SIM domain, which also functions in the recruitment of RAP80-BRCA1 to damage sites^42^,^43^. However, whether deubiquitination of RAP80 affects the binding between SIM and SUMO conjugates needs further investigation.

Posttranslational modification of RAP80 is important for its function in DNA repair and radiosensitivity. For example, phosphorylation of RAP80 on Ser 677 by the Cdk1-cyclin B1 complex is important for RAP80 sensitivity to IR and G2/M checkpoint control^44^. RAP80 was also reported to be phosphorylated by ATM at multiple sites^45^. Moreover, RAP80 is polyubiquitinated and degraded by the anaphase-promoting complex (APC/C)^Cdc20^ or (APC/C)^Cdh1^ in a cell cycle-dependent manner^46^. Here we demonstrate that ubiquitination of RAP80 negatively regulates its focus formation and DDR-related functions but not protein degradation. Ubiquitination was reported to affect multiple cellular signalling in DDR^38^,^47^, including protein degradation and signalling transduction. For example, ubiquitination modification was reported to facilitate DNA damage proteins' recruitment to DSBs through interactions with the ubiquitin binding domain of these proteins, such as RAP80 and 53BP1 (refs 20, 21, 22, 40, 48, 49). However, whether ubiquitination inhibits protein interaction is not clear. A recent study suggested KEAP1 ubiquitinates PALB2 and blocks its interaction with BRCA1 (ref. 50). Here we report another model of regulation for RAP80 by polyubiquitination. We demonstrated that polyubiquitination of RAP80 blocks its interaction with polyubiquitin chain and deubiquitination of RAP80 by USP13 facilitate the interaction between RAP80 and polyubiquitin chain. A previous study on the structure of RAP80 suggested the side chains of Phe 85, Leu 87, Ala 88, Leu 89, Met 91 and aliphatic portions of the Gln 84 side chain in the UIM1 domain of RAP80 and side chains of Leu 109, Leu 110, Ala 113, Ile 114 and aliphatic portions of the side chains of Lys 112 and Glu 116 in the UIM2 domain form a hydrophobic surface to interact with the Ile 44-centred hydrophobic patch of the ubiquitin^51^. Our data showed three lysine sites 75, 90 and 112, which are in or close to the hydrophobic interaction surface, are themselves ubiquitinated, which might in turn interfere with UIM domain's binding to polyubiquitin chain. The deubiquitination is critical for RAP80 binding with polyubiquitin chain. We propose two hypotheses. First, ubiquitination chain conjugated on these three sites may physically block UIM's-ubiquitin binding. Alternatively, ubiquitination may change the UIM's structure and limit the interface in contact with Ub K63 linkage. However, further structural studies are warranted to reveal the underlying mechanism. In addition, the E3 ligase that mediates K75/90/112 ubiquitination is unclear, and remains to be investigated.

Platinum-based agents are important for cancer therapy. Tumour resistance to platinum drugs has become a very challenging problem to overcome. The cytotoxicity of platinum-based agents is dependent on the formation of platinum-DNA adducts, which in turn induce DNA damage. The balance between DNA damage and DNA repair determines whether cancer cells live or die after platinum therapy^52^. To escape killing, tumour cells may evolve to have higher repair efficiency. Recently, PARP inhibitors were utilized to treat BRCA1 and BRCA2 mutation-associated ovarian and breast cancers. However, ovarian or breast cancer cells with intact HR easily develop resistance to PARP inhibitors and often have enhanced HR repair efficiency. Therefore, targeting DNA repair pathways may be a strategy to overcome platinum and PARP inhibitor resistance in tumour^53^. In our study, we demonstrated that USP13 is overexpressed in ovarian cancer cell lines. Depleting or inhibiting USP13 sensitizes ovarian cancer cells but not normal ovarian epithelial cells to cisplatin and PARP inhibitor. This suggests a new mechanism of ovarian cancer chemoresistance and identifies a novel potential drug target in ovarian cancer. The expression of USP13 in cancer is complicated and may be different in different cancers. A previous publication suggests that USP13 regulates PTEN and is downregulated in breast cancer^31^. However, several studies showed that USP13 is overexpressed in other cancers, such as GBM and melanoma^30^,^54^. In our study, both the Oncomine data set (Fig. 6a and Supplementary Fig. 9a) and the TCGA data set (Supplementary Fig. 9b) show the amplification of USP13 gene in ovarian cancer. Most importantly, although BRCA1 deletion and mutation are observed in a subset of ovarian cancers, they are mostly absent in samples with USP13 amplification. In samples with USP13 amplification, most of the BRCA1-A members are expressed normally. All these results support our hypothesis that USP13 amplification in ovarian cancers may confer higher HR activity and resistance to chemotherapy. Targeting USP13 in this subset of cancer may re-sensitize cancer to platinum or PARPi treatment. Finally, considering that USP13 is overexpressed in ovarian cancers, a combination of USP13 inhibitor Spautin-1 and platinum-based therapy or PARP inhibitor may provide a novel approach for ovarian cancer therapy.

## Methods

### Cell culture

HEK293T, U2OS and human ovarian cancer cell lines OVCAR3, SKOV-3 were purchased from ATCC. FU-OV-1 and EFO-27 were purchased from DSMZ. A2780 was kindly provided by Dr Scott Kaufmann (Mayo Clinic). HOSE cells were purchased from ScienCell. The identities of all cell lines were confirmed by the Medical Genome Facility at Mayo Clinic Center (Rochester, MN) using short tandem repeat profiling upon receipt. Periodic Hoechst 33258 staining assays were performed in these cells to detect mycoplasma contamination. HEK293T and U2OS were maintained in RPMI-1640 media with 10% FBS. Human ovarian cancer cell lines OVCAR3, A2780 and SKOV-3 were maintained in RPMI-1640 media supplemented with 10% FBS and 2 mM L-glutamine; FU-OV-1 was maintained in DMEM:F12 with 10% FBS. EFO-27 was maintained in RPMI-1640 media supplemented with 20% FBS, 2 mM L-glutamine, 1x MEM non-essential amino acids, and 1 mM sodium pyruvate. HOSE cell was maintained in Ovarian Epithelial Cell Medium (OEpiCM, Cat. #7311, ScienCell).

### Plasmids and antibodies

HA-FLAG-USP13 was purchased from Addgene (Plasmid #22568, provided by Dr Wade Harper) and subcloned into pGEX-4 T-2 vector (Clontech). USP13 site mutants were generated by site-directed mutagenesis (Stratagene).

The anti-USP13 (GTX118595, dilution: 1:500) and anti-Rad51 (N1C2, dilution: 1:200) antibodies were purchased from Genetex. Anti-Ub (P4D1, dilution: 1:500), anti-RPA32 (9H8, dilution: 1:200) and anti-BRCA1 (D9, dilution: 1:200) antibodies were purchased from Santa Cruz Biotechnology. Anti-γH2AX (05-636, dilution: 1:500), anti-FK2 (04-263, dilution: 1:500) and anti-MDC1 (05-1572, dilution: 1:200) were purchased from Millipore. Anti-RAP80 (A303-763A, dilution: 1:500) and anti-53BP1 (A300-272A, dilution: 1:500) were purchased from Bethyl Laboratories. Anti-FLAG (F1804, dilution: 1:1,000), anti-HA (H9658, dilution: 1:1,000), and anti-β-actin (A1978, dilution: 1:2,000) antibodies were purchased from Sigma. Anti-RNF8 (ab4183, dilution: 1:500) was purchased from Abcam.

### CRISPR/Cas9 knockout

For CRISPR/Cas9 knockout of human USP13 and RAP80 in EFO-27 cells, the following sgRNAs were used. sgUSP13-1: 5′-GACCTGGGCACGCGGATCGT-3′; sgUSP13-2: 5′-GCATGGAGGCGGCAACCAACA-3′. sgRAP80: 5′-ATTGTGATATCCGATAGTGA-3′.

### Co-immunoprecipitation (Co-IP) assay

Cells were lysed with NETN buffer (20 mM Tris-HCl, pH 8.0, 100 mM NaCl, 1 mM EDTA, 0.5% Nonidet P-40) containing 50 mM β-glycerophosphate, 10 mM NaF and 1 mg ml^–1^ each of pepstatin A and aprotinin. Whole cell lysates obtained by centrifugation were incubated with 2 μg of antibody and protein A or protein G-Sepharose beads (Amersham Biosciences) for 4 h at 4 °C. The immunocomplexes were then washed with NETN buffer three times and separated by SDS–polyacrylamide gel electrophoresis. Immunoblotting was performed following standard procedures. The uncropped versions of western blots are shown in Supplementary Figs 11–13.

### Denatured *in vivo* and *in vitro* deubiquitination assay

For the *in vivo* deubiquitination assay, control cells, USP13 knockout cells or USP13 knockout U2OS cells stably expressing HA-USP13 wild-type (WT) or mutant Cys 345 to Ala (CA mutant), were lysed in 120 μl 62.5 mM Tris-HCl (PH 6.8), 2% SDS, 10% glycerol, 20 mM NEM and 1 mM iodoacetamide, boiled for 15 min, diluted 10 times with NETN buffer containing protease inhibitors, 20 mM NEM and 1 mM iodoacetamide and centrifuged to remove cell debris. The cell extracts were subjected to immunoprecipitation with the indicated antibodies and blotted with anti-Ub antibody.

For the preparation of a large amount of ubiquitinated proteins as the substrate for the deubiquitination assay *in vitro*, HEK293T cells were transfected together with the HA-RAP80 and His-Ub expression vectors. Ubiquitinated proteins were purified from the cell extracts with Ni-NTA beads under denatured conditions. After that, the Ub-RAP80 proteins were purified from the eluant with anti-HA-agarose beads in lysis buffer (50 mM Tris-HCl pH 7.8, 137 mM NaCl, 10 mM NaF, 1 mM EDTA, 1% Triton X-100, 0.2% Sarkosyl, 1 mM DTT, 10% glycerol and fresh proteinase inhibitors). The recombinant GST-USP13 and USP13CA were expressed in BL21 cells and purified following standard protocol. For the deubiquitination assay *in vitro*, ubiquitinated proteins were incubated with recombinant USP13 in a deubiquitination buffer (50 mM Tris-HCl pH 8.0, 50 mM NaCl, 1 mM EDTA, 10 mM DTT, 5% glycerol) for 4 h at 30 °C.

### HR assay

We generated control or USP13 knockout U2OS DR-GFP cell lines by CRISPR system using the U2OS DR-GFP cells from Dr Maria Jasin (Memorial Sloan-Kettering Cancer Center). I-SceI expression vector (pCBA-I-SceI) was transfected into the cells. Cells were harvested 2 days after I-SceI transfection and subjected to flow cytometric analysis to examine recombination induced by I-SceI digestion. The parallel transfection with pEGFP-C1 was used to normalize for transfection efficiency.

### Immunofluorescence

To visualize ionizing radiation-induced foci, cells were cultured on coverslips and treated with 2 Gy IR followed by recovery for 1 h or as indicated. Cells were then washed in PBS, incubated in 3% paraformaldehyde for 15 min, and permeabilized in 0.5% Triton-X solution for 5 min at room temperature. Samples were blocked with 5% goat serum and then incubated with primary antibody for 30 min. Samples were washed three times and incubated with secondary antibody for 30 min. Cells were then stained with DAPI to visualize nuclear DNA. The coverslips were mounted onto glass slides with anti-fade solution and visualized using a Nikon eclipse 80i fluorescence microscope. More than 200 cells were counted per experiment.

### Colony formation assay

Cells (500–2,000) were plated in triplicate in each well of six-well plates. After 1 day, cells were randomly divided into treatment groups and exposed to ionizing radiation or treated with the PARP inhibitor, Olaparib, and left for 10–14 days at 37 °C to allow colony formation. Colonies were stained with 5% GIEMSA and counted. Results were normalized to plating efficiencies.

### Tumour xenograft

Experiments were performed under the approval of the Institutional Animal Care and Use Committee at Mayo Clinic (Rochester, MN). Control (ctrl) or USP13 knockout EFO-27 cells were injected subcutaneously and bilaterally into the flanks of 5-week-old female athymic nude NCr nu/nu (NCI/NIH) mice using 19-gauge needles. Each mouse received two injections of a 200 μl mixture of 2 × 10^6^ cells in 100 μl of 1 × PBS and 100 μl of growth factor reduced MATRIGEL (BD Biosciences). Mice bearing tumours of 150–200 mm^3^ were divided into two groups by stratified randomization: vehicle control group (10% DMSO with 10% 2-hydroxypropyl-beta-cyclodextrin daily) and Olaparib group (50 mg kg^–1^ daily). USP13 high expression EFO-27 cells were injected as previously described and mice bearing tumours of 150–200 mm^3^ were divided into four groups by stratified randomization: vehicle control group (10% DMSO with 10% 2-hydroxypropyl-beta-cyclodextrin daily), Olaparib group (50 mg kg^–1^ daily), Spautin-1 group (20 mg kg^–1^ daily) and Spautin-1(20 mg kg^–1^ daily) together with Olaparib (50 mg kg^–1^ daily) group. Each group contained five nude mice. Tumour volume was subsequently measured every 4 days using calipers and tumour volume was calculated as 0.5 × L × H × W. Mice were killed for tumour dissection on day 28 after the start of Olaparib treatment. Data were analysed using analysis of variance test. A *P* value <0.05 was considered significant. Mice were subjected to euthanasia if they displayed pain or distress, such as lethargy, lying down, not eating or drinking, weight loss greater than 10% body weight, or difficulty breathing. According to the blinding procedures, two people as a group performed all the mice experiments. One person performed the experiments and another one totally blinded to the experiment group measured the tumour volume and weight and analysed the data.

### Statistics

For cell survival assay and HR assay, data are presented as the mean±s.e.m. of three independent experiments. For foci formation assay, data are presented as the mean±s.e.m. of three independent experiments. More than 200 cells were counted per experiment. For animal study, data are represented as the mean±s.d. of five mice. Statistical analyses were performed with the student's *t*-test or analysis of variance test. Statistical significance is represented in figures by: *P<0.05; **P<0.01.

### Data availability

All data generated or analysed during this study are available within the article and Supplementary Files, or available from the authors upon request.

## Additional information

**How to cite this article:** Li, Y. *et al*. USP13 regulates the RAP80-BRCA1 complex dependent DNA damage response. *Nat. Commun.* **8,** 15752 doi: 10.1038/ncomms15752 (2017).

**Publisher's note:** Springer Nature remains neutral with regard to jurisdictional claims in published maps and institutional affiliations.

## Supplementary Material

Supplementary Information Supplementary Figures
