# Supplementary material for: USP13 regulates the RAP80-BRCA1 complex dependent DNA damage response
Source: Nat Commun. 2017 Jun 1;8:15752. doi: 10.1038/ncomms15752 (PMC5461494; doi:10.1038/ncomms15752)
Supplement: Supplementary Information — Supplementary Figures [file ncomms15752-s1.pdf]

Supplementary Figure 1

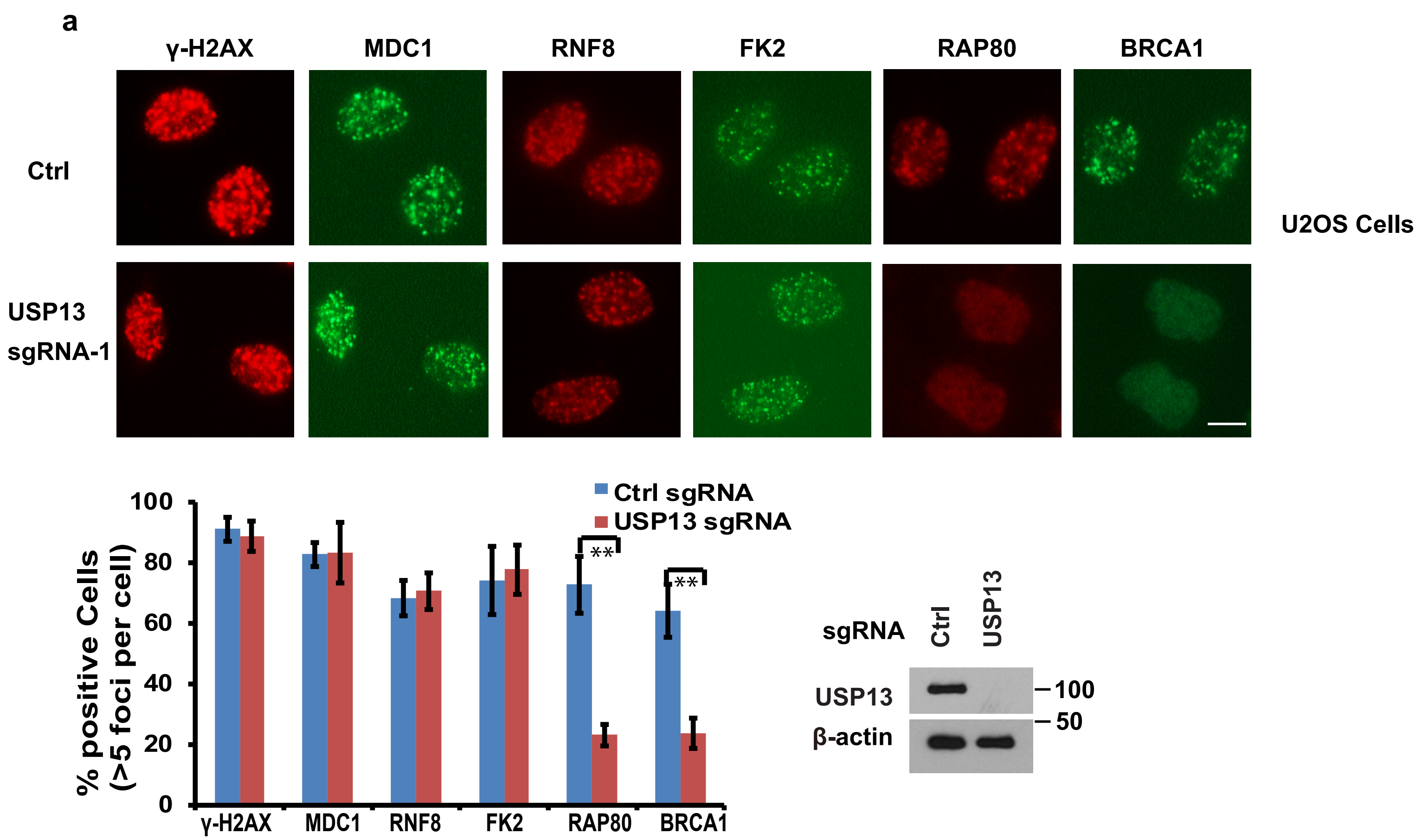

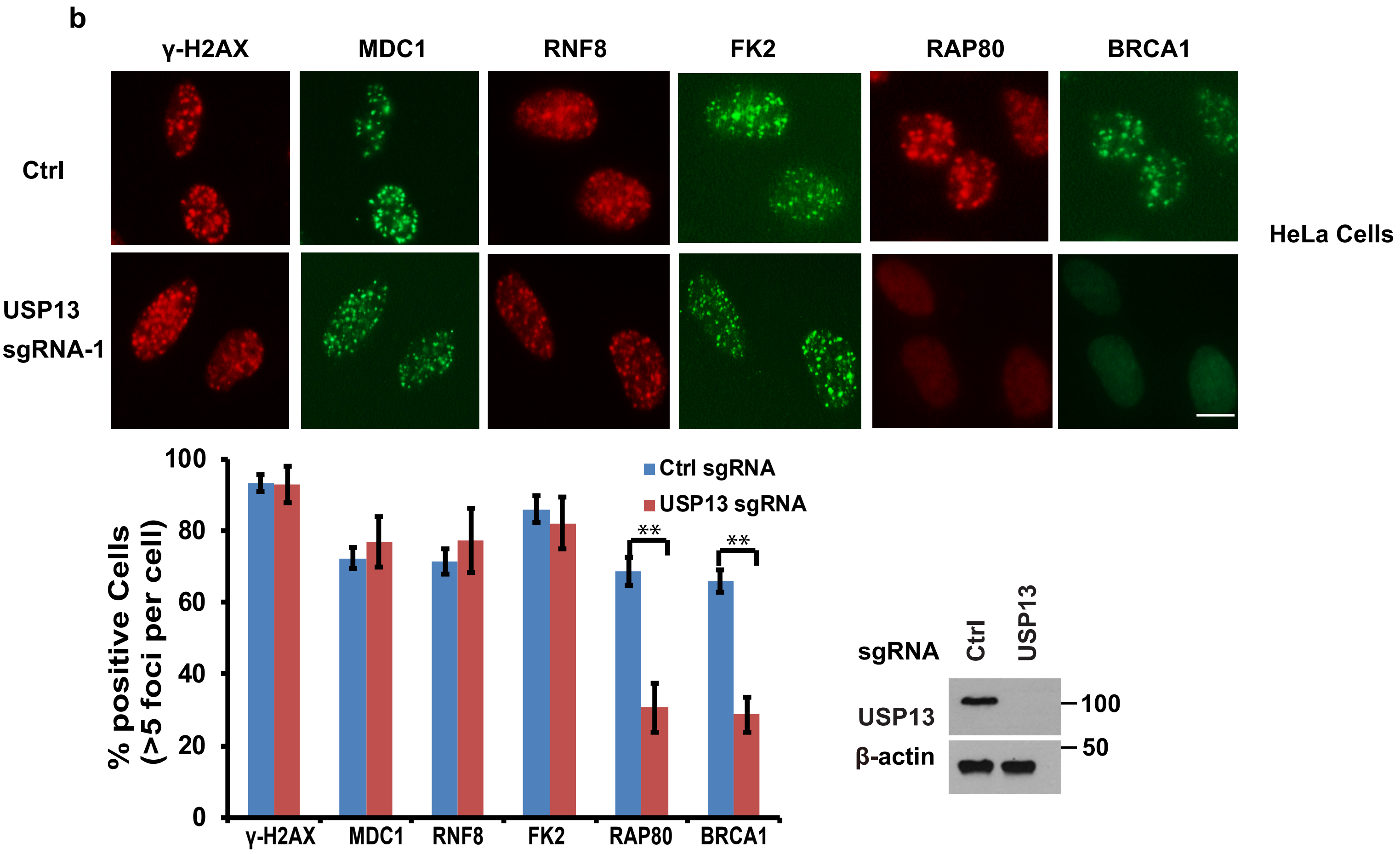

Supplementary Figure 1. USP13 regulates RAP80-BRCA1 foci formation in response to cisplatin. (a-b) Control or USP13 knockout U2OS (a) or HeLa (b) cells were treated with cisplatin and foci formation of the indicated factors were detected by immunofluorescence with indicated antibodies. Representative images are shown in the upper panels. Scale bar, 10 μm. Quantification of the percentage of cells displaying foci formation is shown in the lower left panels. USP13 expression in these cells were examined by western blot and is shown in the lower right panel. Error bars represent SEM from three independent experiments. **P < 0.01. >200 cells were counted per experiment. Statistical analyses were performed with the ANOVA.

Supplementary Figure 2

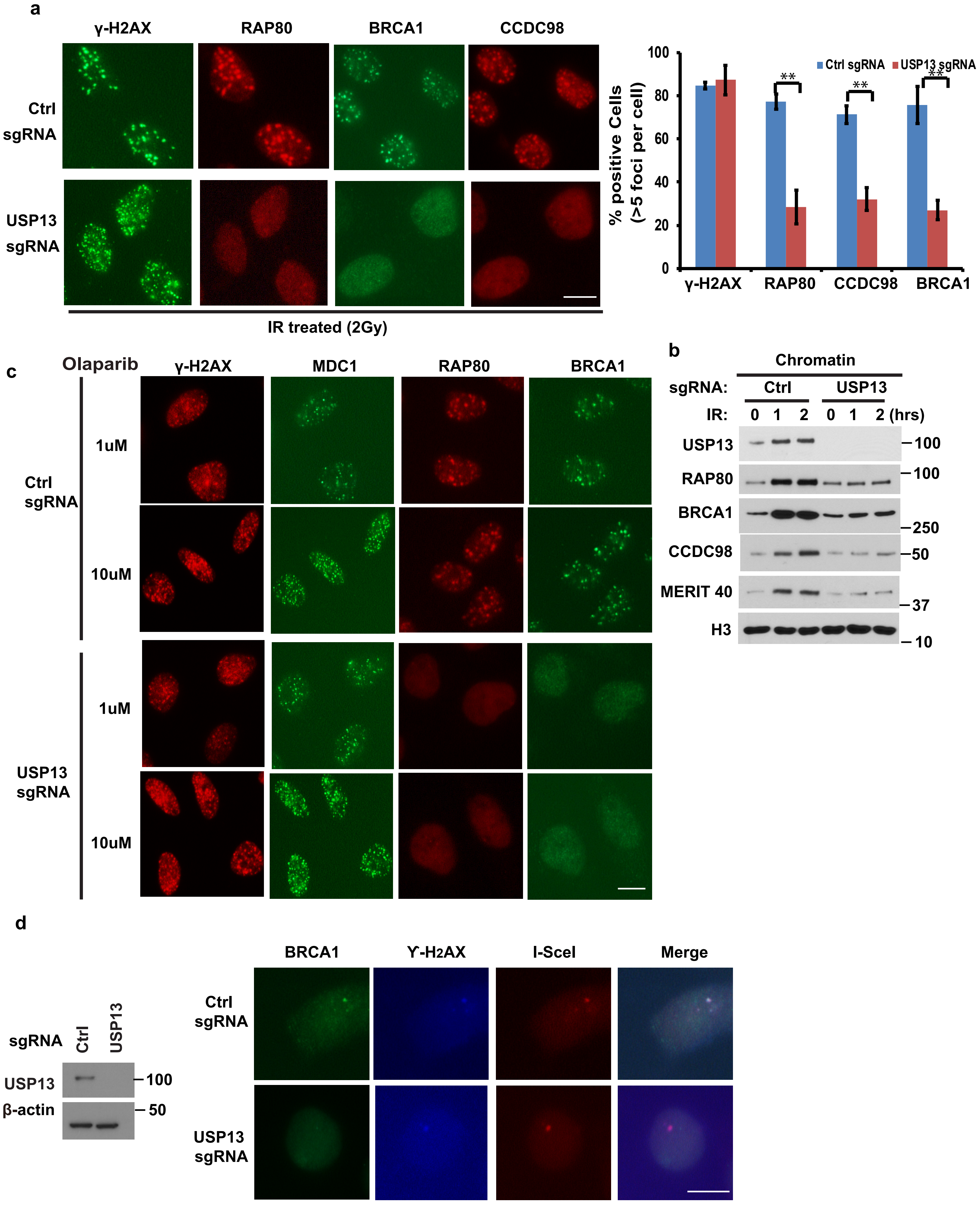

Supplementary Figure 2. USP13 regulates BRCA1-A complex recruitment to DSBs. (a) Control or USP13 knockout EFO-27 cells were treated with IR (2Gy) and foci formation of the indicated factors were detected by immunofluorescence with indicated antibodies. Representative images are shown in the left panels. Quantification of the percentage of cells displaying foci formation is shown in the right panels. Scale bar, 10 μm. Error bars represent SEM from three independent experiments. **P < 0.01. >200 cells were counted per experiment. Statistical analyses were performed with the ANOVA. (b) Control or USP13 knockout EFO-27 cells were treated with IR (2Gy). Whole cell lysates and chromatin fractions were prepared and blotted with the indicated antibodies. (c) Control or USP13 knockout EFO-27 cells were treated with olaparib at indicated concentrations for 6 hr. Foci formation of the indicated factors was detected by immunofluorescence with indicated antibodies. Scale bar, 10 μm. (d) Co-localization of BRCA1 with γ-H2AX at DSB site created by I-SceI was examined in control or USP13 knockout cells. Scale bar, 10 μm. USP13 expression in these cells was examined by western blot and is shown in the panel to the left.

Supplementary Figure 3

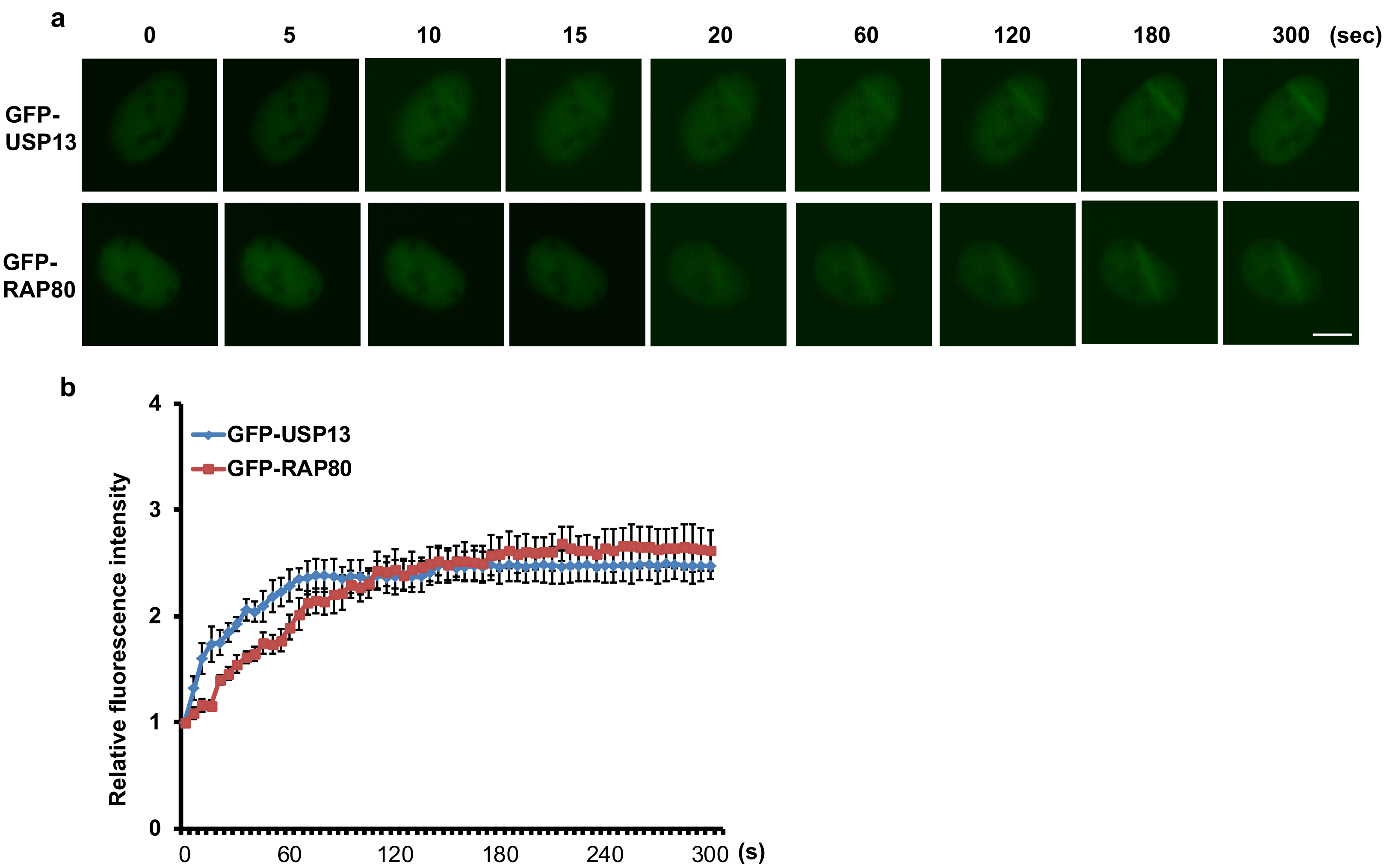

Supplementary Figure 3. Recruitment kinetics of USP13 and RAP80. (a-b) Cells expressing GFP-USP13 or GFP-RAP80 were subjected to laser microirradiation. Laser stripes were examined at the indicated time points. Scale bar, 10 μm. The intensity of each laser stripe at each time point was determined by averaging values from 10 cells and is graphed in (b).

Supplementarg Figure 4

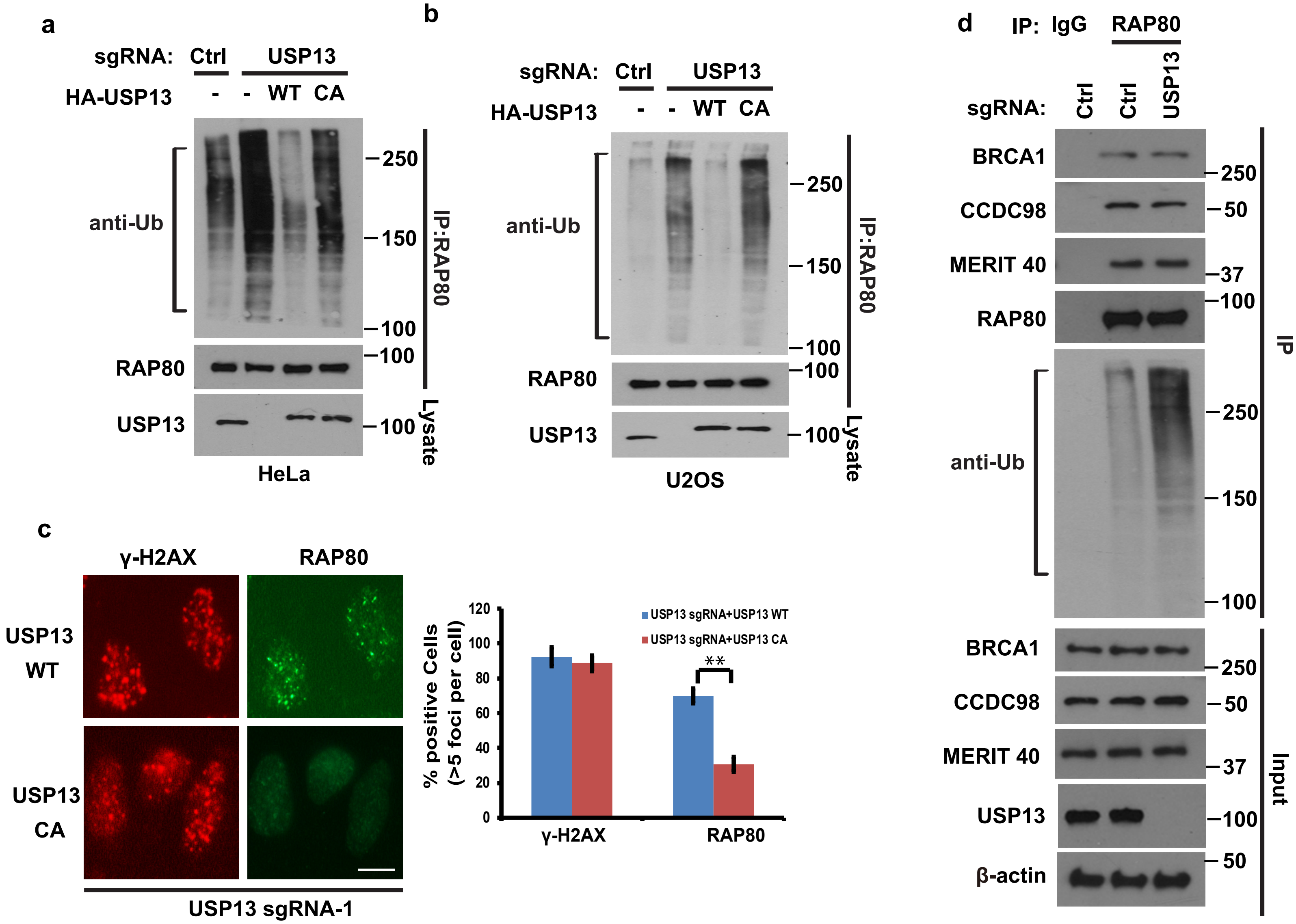

Supplementary Figure 4. Deubiquitination of RAP80 by USP13 affects RAP80 foci formation but not BRCA1-A complex stabilization. (a-b) HeLa (a) or U2OS (b) cells stably expressing control, USP13 sgRNA, or USP13 sgRNA with the indicated constructs, were lysed under denaturing conditions and RAP80 was immunoprecipitated. Blots were probed with the indicated antibodies. (c) EFO-27 cells stably expressing USP13 sgRNA with the indicated constructs were treated with cisplatin and foci formation of the indicated factors were detected by immunofluorescence with indicated antibodies. Representative images are shown in the left panel. Scale bar, 10 μm. Quantification of the percentage of cells displaying foci formation is shown in the right panel. Error bars represent SEM from three independent experiments. **$P < 0.01$. >200 cells were counted per experiment. Statistical analyses were performed with the Student's t-test. (d) Cell lysates from control (Ctrl) or USP13 knockout EFO-27 cells were subjected to immunoprecipitation with control IgG or RAP80 antibodies. The western blots were then blotted with the indicated antibodies.

Supplementary Figure 5

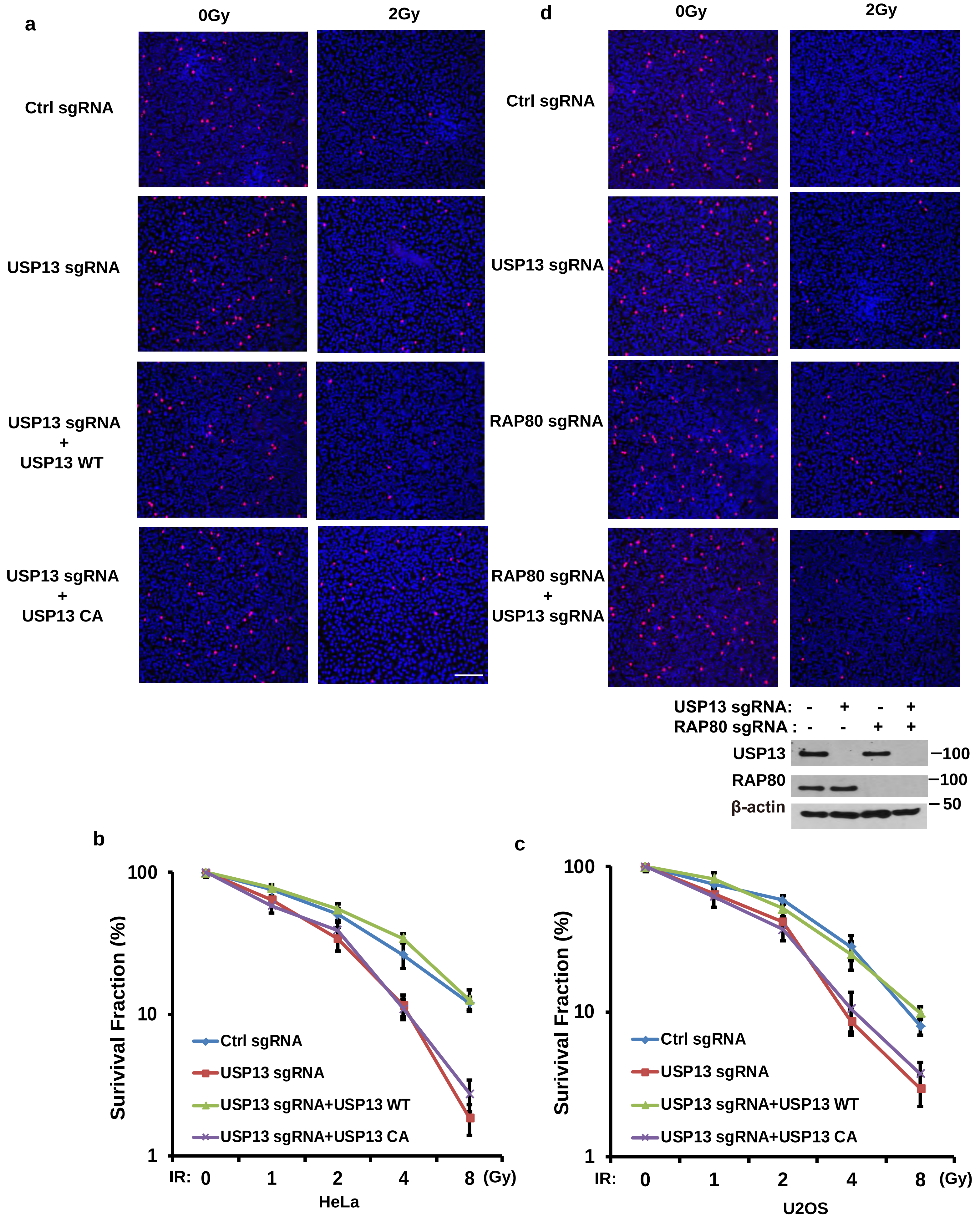

Supplementary Figure 5. USP13 regulates G2/M checkpoint and radiosensitivity. (a) The representative data for Fig 3a. Scale bar, 500 μm. (b-c) HeLa (b) or U2OS (c) cells stably expressing control, USP13 sgRNA, or USP13 sgRNA with the indicated constructs were generated. Sensitivity of these cells to IR was assessed using colony formation assay. Error bars represent SEM from three independent experiments. (d) The representative data for Fig 3d. Scale bar, 500 μm. USP13 and RAP80 expression in these cells were examined by western blot and is shown in the lower panel.

Supplementary Figure 6

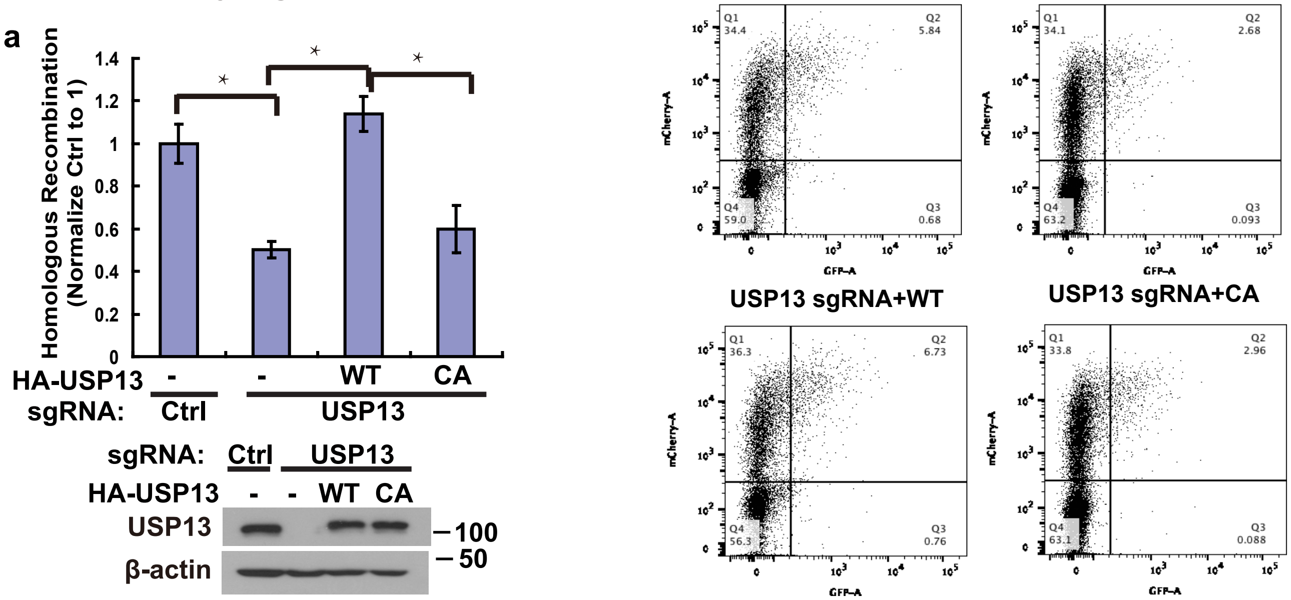

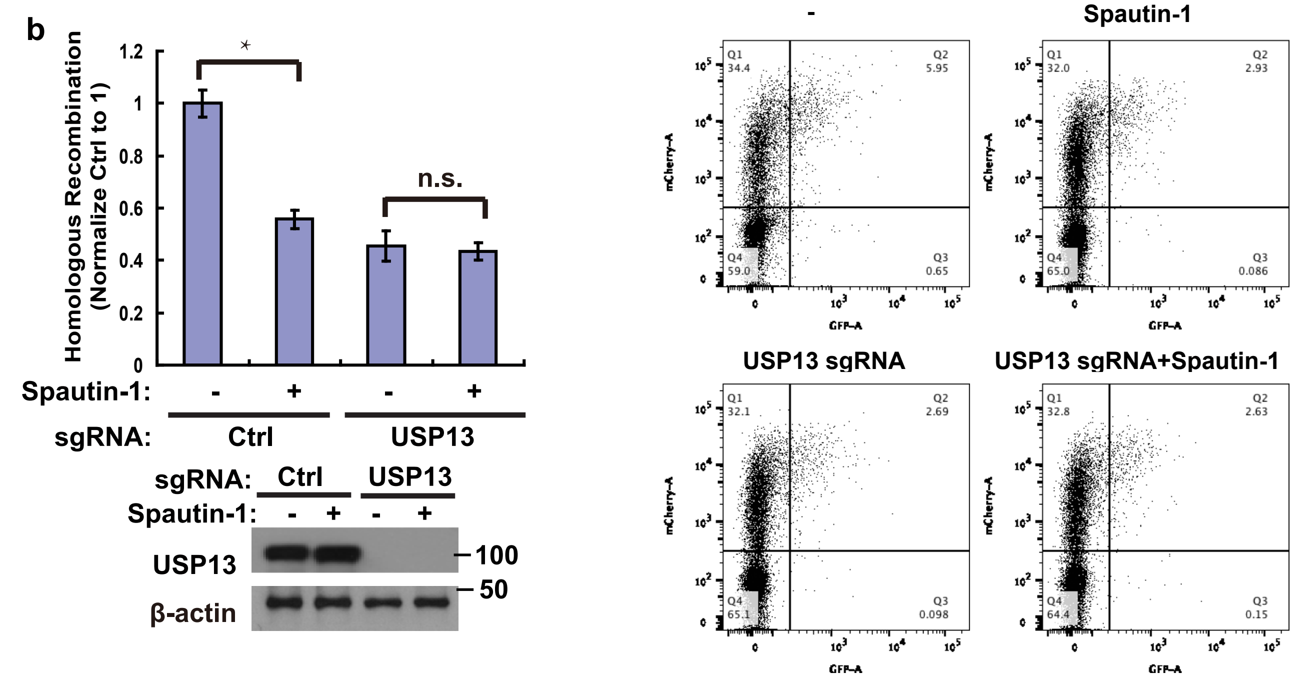

Supplementary Figure 6. USP13 regulates HR. (a) HR-mediated DSB repair capacity of control, USP13 knockout, and USP13 knockout cells stably expressing the indicated constructs were assessed using a reporter system. (b) HR-mediated DSB repair capacity of control and USP13 knockout cells treated with or without Spautin-1 was assessed using a reporter system. Error bars represent SEM from three independent experiments. *P < 0.05. Statistical analyses were performed with the ANOVA.

Supplementary Figure 7

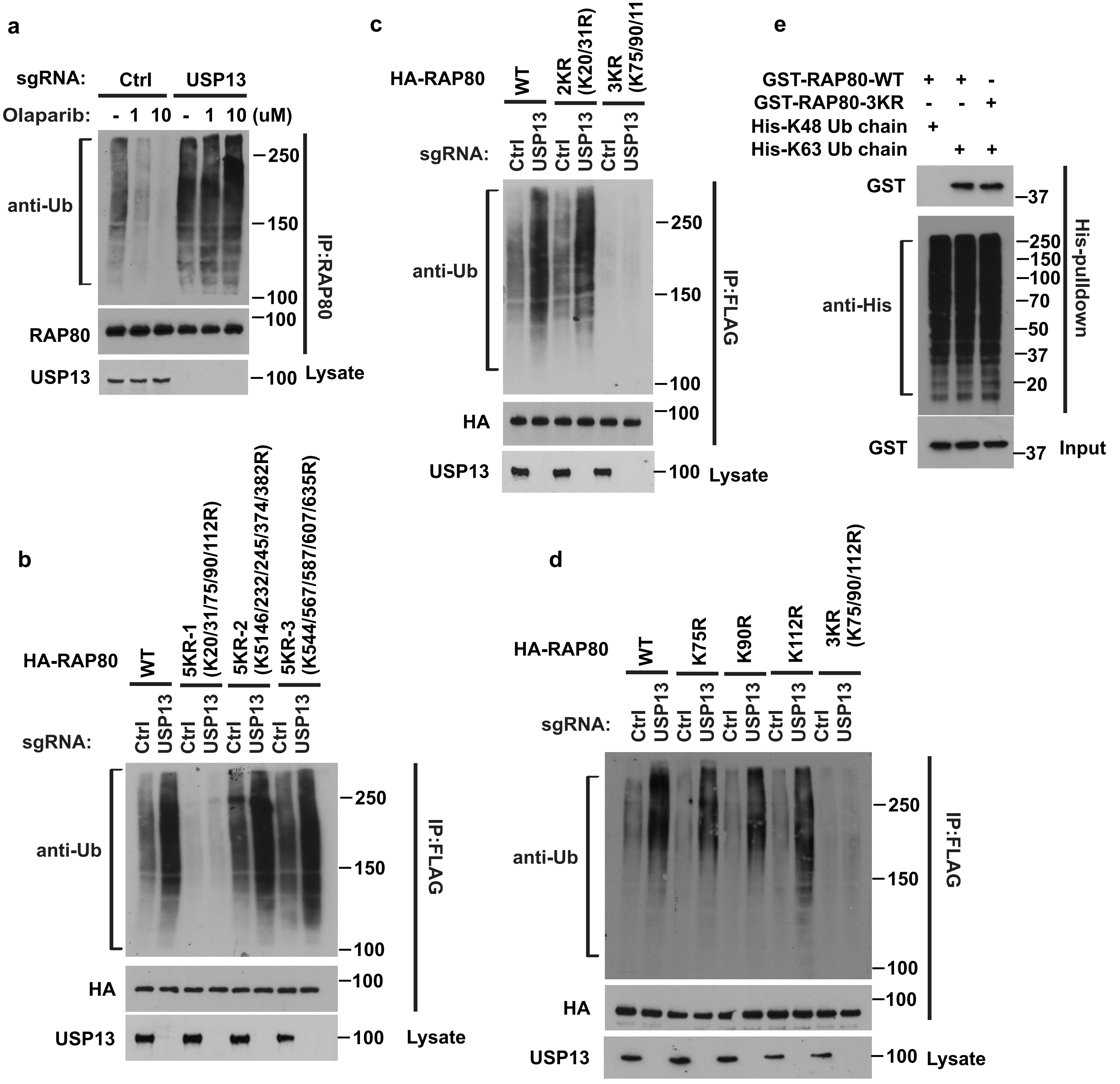

Supplementary Figure 7. Identification of the RAP80 deubiquitination sites targeted by USP13. (a) Control and USP13 knockout cells treated with olaparib at different concentrations were lysed under denaturing conditions and RAP80 was immunoprecipitated. Blots were probed with the indicated antibodies. (b-d) Control and USP13 knockout cells were transfected with indicated constructs. Cells were lysed under denaturing conditions and HA-RAP80 was immunoprecipitated. Blots were probed with the indicated antibodies. (e) GST-RAP80 WT and 3KR mutant proteins were subjected to pull down assay by incubating with indicated His-Ub chains conjugated with Ni-NTA beads. After washing, proteins bound on beads were subjected to western blot with indicated antibodies.

Supplementary Figure 8

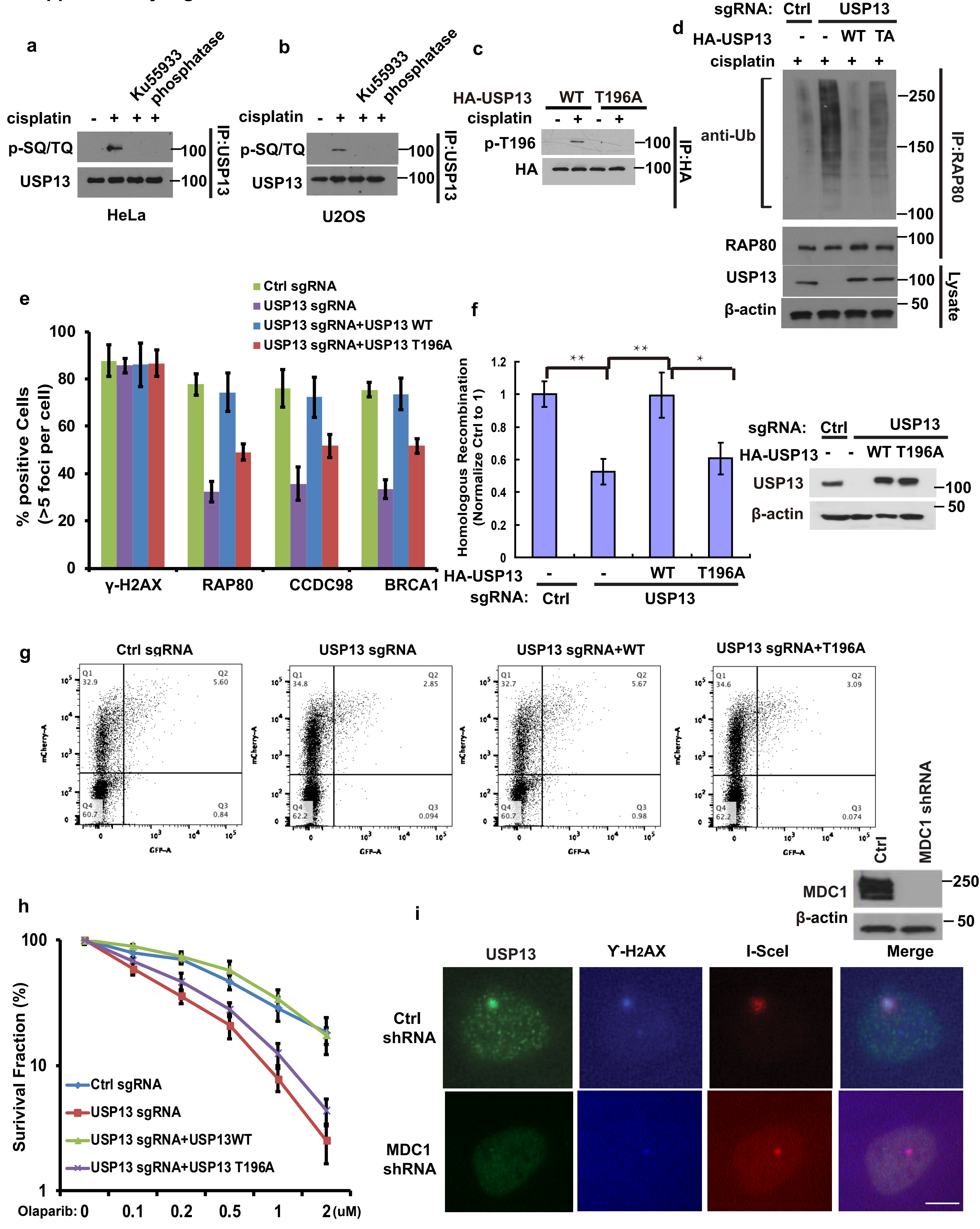

Supplementary Figure 8. Phosphorylation of USP13 regulates DDR. (a-b) HeLa (a) or U2OS (b) cells were pretreated with DMSO or 25μM Ku55933 for 2 hrs following which they were left untreated or treated with cisplatin. After an additional 1 hr, USP13 was immunoprecipitated, left untreated or treated with phosphatase, and immunoblotted with phospho-SQ/TQ (pSQ/TQ) antibody. (c) HEK293T cells transfected with HA-USP13 WT or T196A mutant were left untreated or treated with cisplatin. HA-USP13 was immunoprecipitated and immunoblotted with T196 phospho-specific antibody. (d) Control and USP13 knockout cells were transfected with the indicated constructs. Cells were lysed under denaturing conditions and RAP80 was immunoprecipitated. Blots were probed with the indicated antibodies. (e) EFO-27 cells stably expressing Ctrl, USP13 sgRNA or USP13 sgRNA with the indicated constructs were treated with cisplatin and foci formation of the indicated factors were detected by immunofluorescence with the indicated antibodies. Error bars represent SEM from three independent experiments. >200 cells were counted per experiment. (f-g) Control, USP13 knockout, and USP13 knockout cells stably expressing the indicated constructs were subjected to DR-GFP based HR assay. Error bars represent SEM from three independent experiments. $^{**}P < 0.01$, $^{*}P<0.05$. Statistical analyses were performed with the ANOVA. USP13 expression is shown in the right panel. The representative FACS data is shown in (g). (h) Control, USP13 knockout, and USP13 knockout cells stably expressing the indicated constructs were subjected to colony formation assay to assess the sensitivity of cells to olaparib. Error bars represent SEM from three independent experiments. (i) Co-localization of USP13 with γ-H2AX at DSB site created by I-SceI was examined in control or MDC1 knockdown cells. Scale bar, 5 μm. MDC1 expression in these cells was examined by western blot and is shown in the right upper panel.

Supplementary Figure 9

a

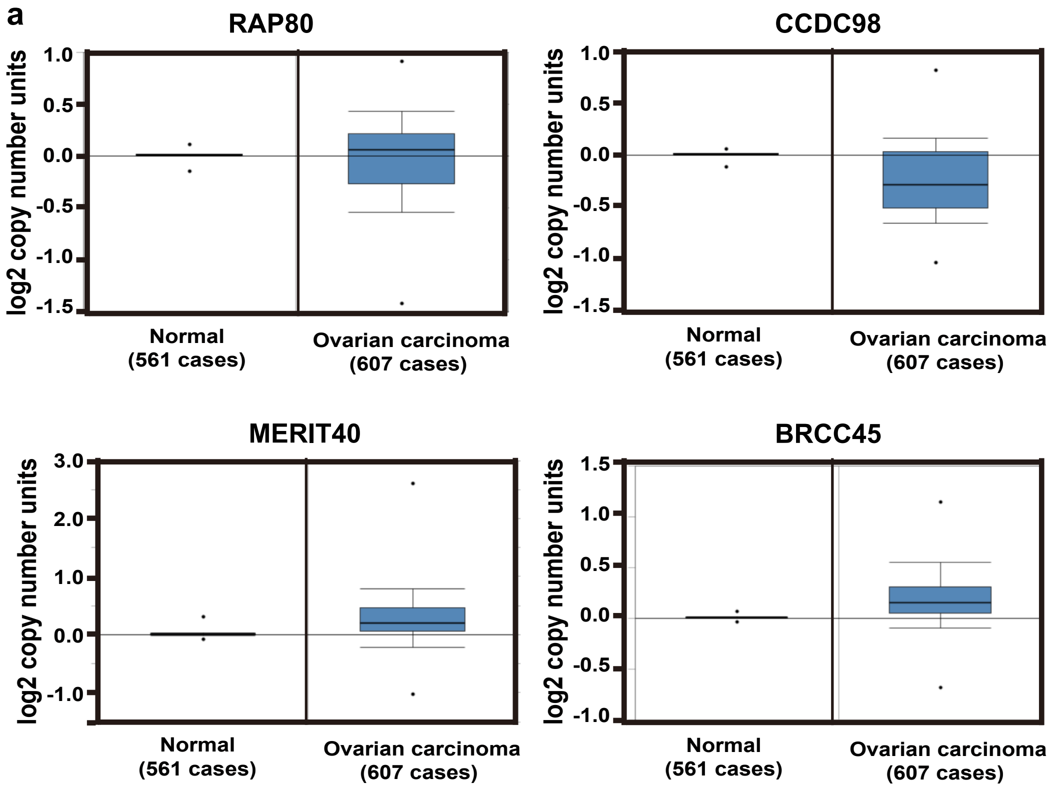

b

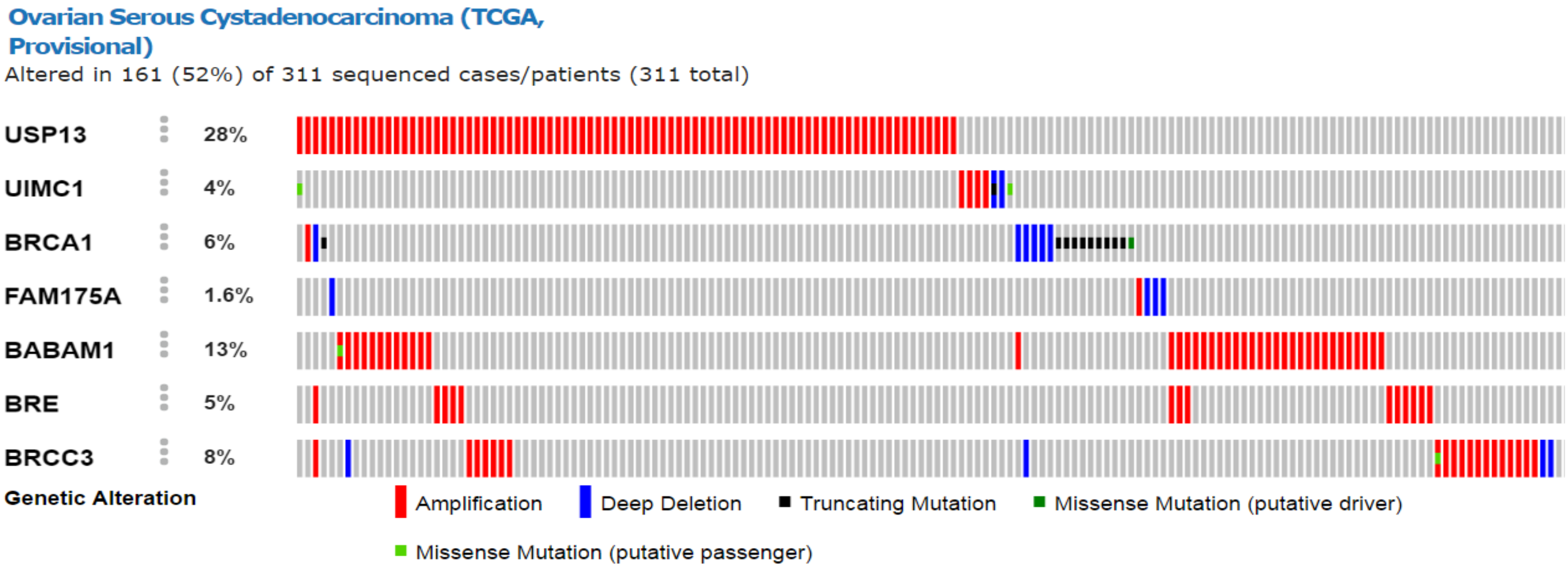

Supplementary Figure 9. The expression of USP13 in ovarian cancer. (a) The expression of BRCA1-A complex in normal and ovarian carcinoma (Oncomine data). (b) Gene copy-number and mutation data of USP13 and BRCA1-A complex in ovarian cancer (TCGA data).

Supplementary Figure 10

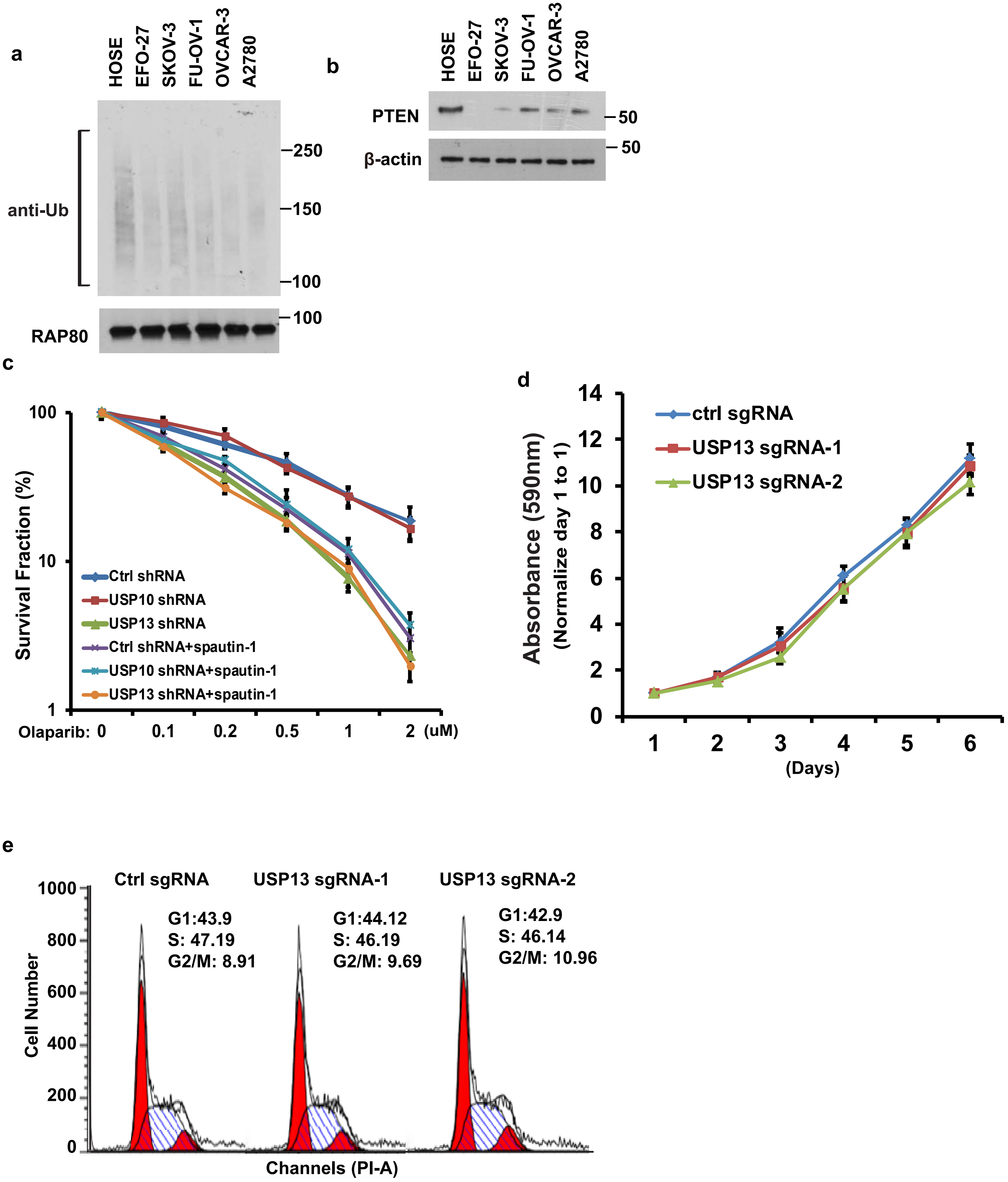

Supplementary Figure 10. USP13 regulates chemoresistance in ovarian cancer. (a) The human ovarian epithelial cell line (HOSE) and ovarian carcinoma cell lines were lysed under denaturing conditions and RAP80 was immunoprecipitated. Blots were probed with the indicated antibodies. (b) Expression of PTEN in human ovarian epithelial cell line and ovarian carcinoma cell lines. (c) Control, USP13 knockdown, and USP10 knockdown cells were subjected to colony formation assay to assess the sensitivity of cells to olaparib or olaparib with spautin-1. (d) Growth curves of EFO-27 cells stably expressed ctrl or USP13 sgRNAs were measured by MTS assay. (c-d) Error bars represent SEM from three independent experiments. (e) Cell cycle profile of EFO-27 cell stably expressed ctrl or USP13 sgRNAs.

Supplementary Figure 11

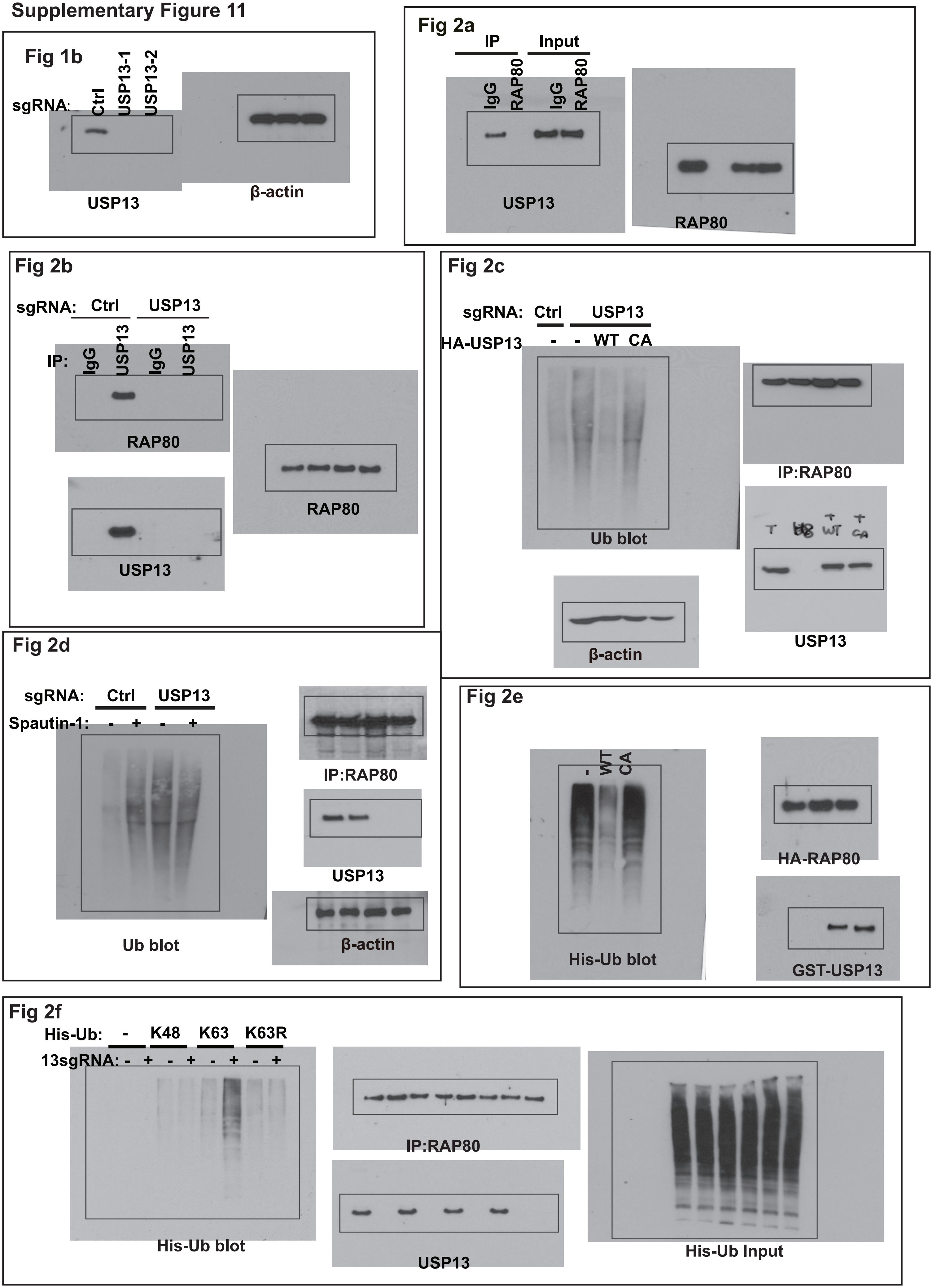

Supplementary Figure 11. **Original scan of the blots presented in the main text. Related to Figure 1 and 2.**

Supplementary Figure 12

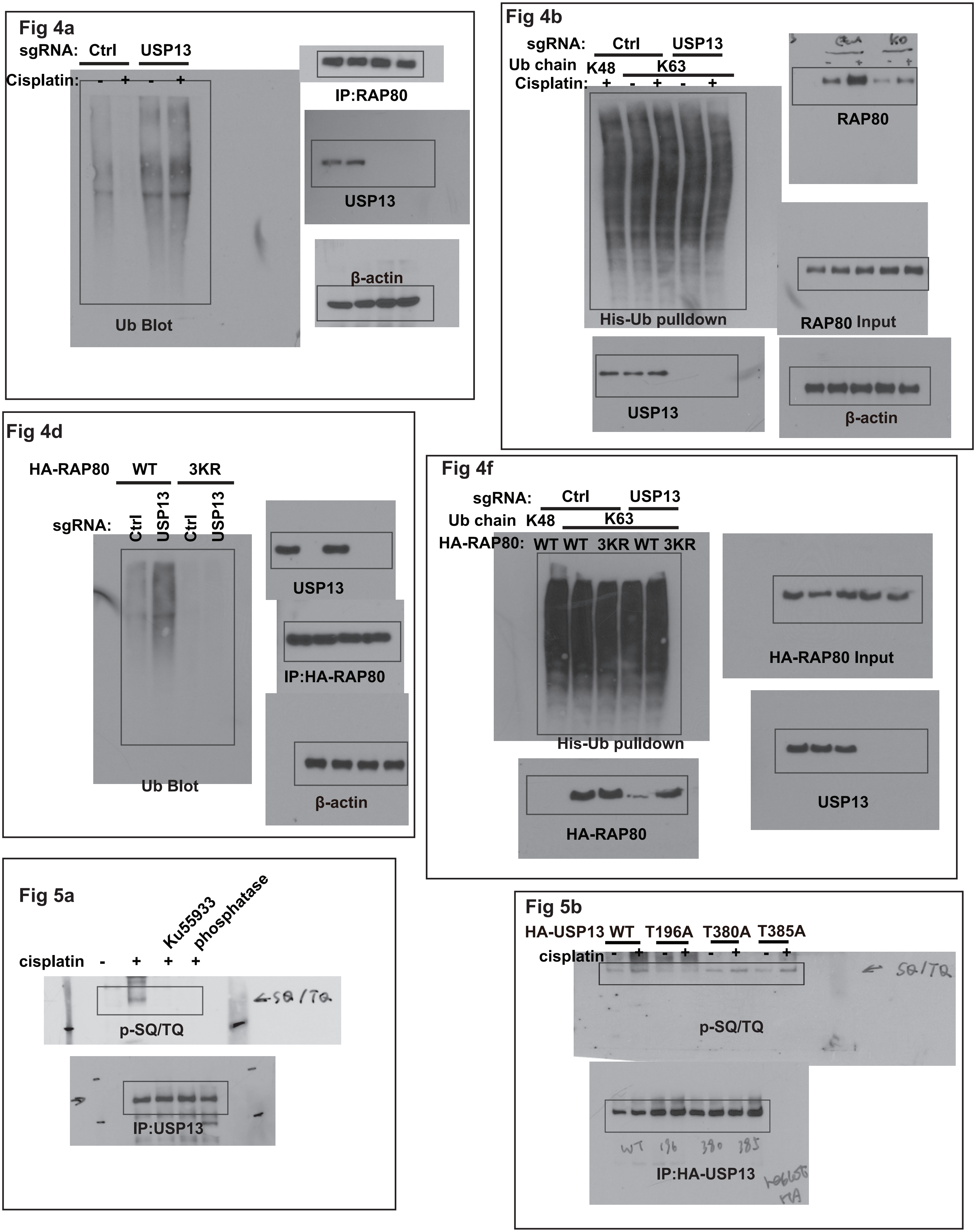

Supplementary Figure 12. Original scan of the blots presented in the main text. Related to Figure 4 and 5.

Supplementary Figure 13

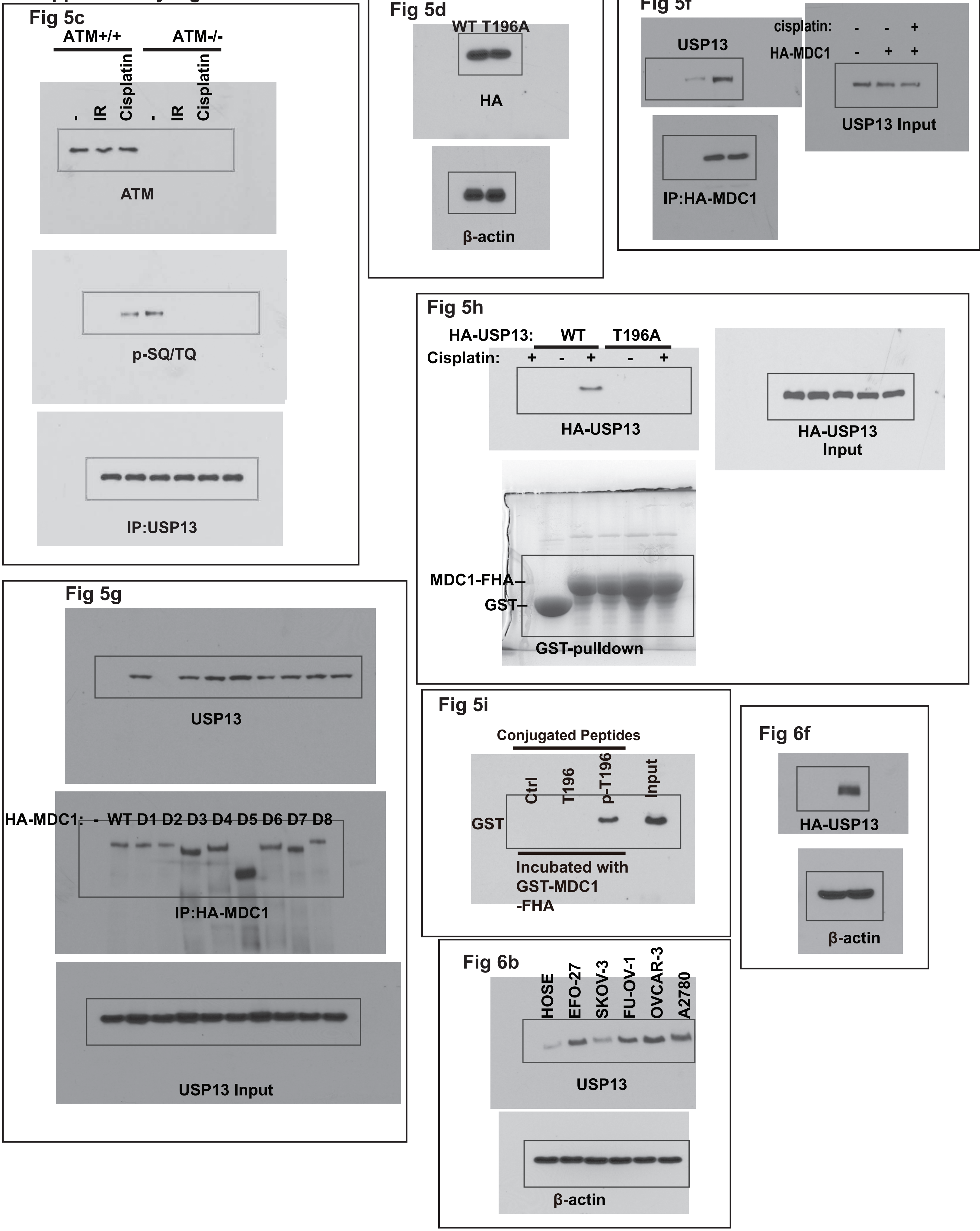

Supplementary Figure 13. **Original scan of the blots presented in the main text. Related to Figure 5 and 6.**
